# Nutritional status, clinical burden, and healthcare utilization among pediatric outpatients with congenital heart disease: A retrospective cross-sectional study from Indonesia

**DOI:** 10.1371/journal.pone.0351014

**Published:** 2026-10-08

**Authors:** Putri Amelia, Lourents Christian Dika Sahertian, Rizky Adriansyah, Johnson Kannady

**Affiliations:** 1 Department of Pediatrics, Faculty of Medicine, Universitas Sumatera Utara, Medan, Indonesia; 2 Department of Pediatrics, Adam Malik Hospital, Medan, Indonesia; 3 Center of Evidence-Based Cardiology, Universitas Sumatera Utara, Medan, Indonesia; Mansoura University Faculty of Medicine, EGYPT

## Abstract

Congenital heart disease contributes substantially to chronic morbidity, growth impairment, and repeated healthcare utilization among children. Evidence regarding nutritional burden and outpatient healthcare patterns among pediatric patients with congenital heart disease in Indonesia remains limited. This study aimed to evaluate clinical characteristics, nutritional status, healthcare utilization, and factors associated with malnutrition among pediatric outpatients with congenital heart disease at Adam Malik General Hospital, Indonesia. A retrospective observational study was conducted using medical records of pediatric outpatients treated between January and December 2024. Demographic characteristics, cardiac diagnoses, nutritional status, complications, and outpatient visit history were analyzed. Logistic regression analysis was performed to identify factors associated with malnutrition. A total of 606 pediatric outpatients were included. Non cyanotic congenital heart disease predominated the cohort, with ventricular septal defect representing the most common diagnosis followed by patent ductus arteriosus and atrial septal defect. Nearly half of all patients demonstrated underweight or severe underweight nutritional status, while pulmonary hypertension emerged as the most frequent complication. Younger pediatric age groups and higher cumulative clinical burden were independently associated with greater odds of malnutrition. Children with congenital heart disease at this tertiary referral center carried a substantial nutritional and clinical burden. Routine nutritional screening, particularly among younger children and those with greater clinical burden may help identify patients who warrant closer nutritional assessment and multidisciplinary follow-up.

## Introduction

Congenital heart disease (CHD) is the most common congenital anomaly in children and remains an important cause of pediatric morbidity and mortality worldwide [1,2]. Although survival has improved with advances in diagnosis and treatment, children with CHD in low and middle income countries still frequently experience delayed diagnosis, limited access to specialized care, and chronic hemodynamic stress that may impair growth and nutritional status [3–6]. Malnutrition is a clinically important comorbidity in this population because it affects immune function, neurodevelopment, surgical readiness, and long-term outcomes [3,4,7].

In addition to nutritional impairment, children with CHD often require repeated outpatient follo – up, reflecting the chronic burden of their disease [8]. However, data from Indonesia remain limited, particularly regarding the relationship between nutritional status, clinical burden, and healthcare utilization in pediatric outpatient settings. Adam Malik General Hospital is a major tertiary referral center in North Sumatra that receives pediatric cardiology referrals from multiple districts across western Indonesia. Therefore, this study aimed to evaluate the clinical characteristics, nutritional status, healthcare utilization, and factors associated with malnutrition among pediatric outpatients with CHD at Adam Malik General Hospital in 2024.

## Methods

### Study design and setting

This study used a retrospective observational design based on secondary data obtained from medical records of pediatric outpatients with congenital heart disease at Adam Malik General Hospital, Medan, North Sumatra, Indonesia. Adam Malik General Hospital functions as a tertiary referral center for pediatric cardiology services in western Indonesia and receives referrals from multiple districts across North Sumatra and surrounding provinces. Data collection covered outpatient visits recorded between January 1 and December 31, 2024.

### Study population and sampling

The study population consisted of all pediatric patients diagnosed with congenital heart disease who attended the pediatric outpatient clinic during the study period. A total sampling approach was applied to include all eligible patients who fulfilled the study criteria.

Inclusion criteria consisted of pediatric patients aged 0–18 years with a confirmed diagnosis of congenital heart disease documented by pediatric cardiologists based on transthoracic echocardiography in the medical record. Patients were included only when demographic information, congenital heart disease classification, nutritional assessment, and outpatient visit history were available.

Patients with incomplete core demographic data or unclear congenital heart disease diagnosis were excluded from the analysis.

### Variables and operational definitions

The primary outcome of this study was malnutrition. Nutritional status was obtained from medical records, where anthropometric assessments had been routinely performed during outpatient visits according to age appropriate growth standards. For children aged <5 years, nutritional status was assessed using the World Health Organization (WHO) Child Growth Standards (2006) based on body mass index (BMI)-for-age z-scores. Underweight was defined as a BMI-for-age z-score between −3 SD and <−2 SD, whereas severe underweight was defined as a BMI-for-age z-score <−3 SD. For children aged ≥5 years, nutritional status was assessed using the Centers for Disease Control and Prevention (CDC) Growth Charts based on BMI-for-age percentiles routinely applied in the institution. Underweight was defined as a BMI-for-age percentile below the 5th percentile. Patients with normal nutritional status served as the reference group. For regression analyses, patients classified as underweight or severe underweight were combined into a single malnutrition category.

Congenital heart disease (CHD) was classified according to physiological status as cyanotic or non-cyanotic CHD based on the documented echocardiographic diagnosis. Individual cardiac lesions, including atrial septal defect, ventricular septal defect, patent ductus arteriosus, pulmonary stenosis, coarctation of the aorta, tetralogy of Fallot, and transposition of the great arteries, were also recorded and summarized descriptively. Physiological classification was selected for multivariable analysis because detailed lesion-specific hemodynamic parameters, including shunt magnitude, ventricular function, and pressure gradients, were not consistently available in the retrospective medical records.

Demographic variables included sex and age group. Age categories consisted of neonates, infants, young toddlers, toddlers, preschool children, school aged children, and adolescents. Because of sparse observations in the extreme age categories, neonatal and adolescent groups were combined in the primary multivariable analysis to improve model stability.

Clinical variables included dyspnea, poor feeding, easy fatigability, cyanosis, poor weight gain, pulmonary hypertension, respiratory tract infection, heart failure, failure to thrive, and Eisenmenger syndrome. Healthcare utilization was assessed using the number of outpatient visits recorded during the study period.

A cumulative burden score was developed as an investigator designed exploratory composite measure to summarize overall clinical burden. The score was calculated as the total number of documented symptoms and complications recorded for each patient during the study period, with higher scores indicating greater cumulative clinical burden. It was used solely for exploratory analyses within the present study because this composite measure has not undergone formal validation.

### Data collection procedure

Medical records were reviewed systematically using hospital registration databases and outpatient cardiology records. Eligible patients were identified from the hospital outpatient registry using congenital heart disease diagnostic codes recorded in the electronic medical record system and subsequently verified through transthoracic echocardiographic documentation. The diagnosis of congenital heart disease was established by pediatric cardiologists according to standard echocardiographic assessment documented in the medical record. Nutritional status assessments were routinely performed during outpatient visits using anthropometric measurements obtained by trained clinical staff as part of standard pediatric care and interpreted according to the institution’s established growth assessment procedures. Relevant demographic, clinical, nutritional, and healthcare utilization variables were subsequently extracted and entered into a structured data collection form. Data cleaning and verification were performed before statistical analysis to reduce entry inconsistencies and duplicate records.

### Missing data handling

Data completeness was evaluated for all study variables before analysis. Demographic variables, congenital heart disease classification, nutritional status, and outpatient visit history were complete. Symptom and complication variables showed substantial missingness, as summarized in S1 Table. Complete case analysis was applied because of missing data in covariates included in the regression model. Therefore, only patients with complete data for all variables included in the final multivariable model were retained for regression analysis. No imputation was performed because the retrospective nature of the dataset and the pattern of missingness did not support reliable imputation.

Missing clinical documentation was considered likely related to inconsistent recording practices rather than true biological absence of symptoms or complications. No imputation procedures were performed because of the retrospective nature of the dataset and the non random pattern of missingness. Complete case analysis was therefore applied for regression modeling.

### Statistical analysis

Statistical analyses were performed using IBM SPSS Statistics and R statistical software. Categorical variables were summarized as frequencies and percentages, whereas continuous variables were presented as mean ± standard deviation or median (minimum–maximum), as appropriate according to data distribution.

Univariate analyses were conducted to describe demographic characteristics, CHD subtype distribution, nutritional status, clinical manifestations, complications, and healthcare utilization patterns.

Factors associated with malnutrition were evaluated using logistic regression analysis. Univariable logistic regression was initially performed to estimate crude odds ratios (ORs) and identify candidate variables. Variables considered clinically relevant or demonstrating statistical association were subsequently entered into the multivariable logistic regression model to estimate adjusted ORs with 95% confidence intervals (CIs).

The primary multivariable model included CHD physiological classification (cyanotic versus non cyanotic), sex, age group, and cumulative burden score as prespecified covariates. Odds ratios (ORs) greater than 1 indicated increased odds of malnutrition, and statistical significance was defined as a two sided p-value <0.05.

Sensitivity analysis was performed using the original non collapsed age group classification to evaluate the robustness of the primary model. Multicollinearity was assessed using generalized variance inflation factor (GVIF) values. Model performance was evaluated using the Akaike information criterion (AIC), Bayesian information criterion (BIC), Tjur’s R^2^, root mean square error (RMSE), logarithmic score (log-loss), and proportion correctly predicted (PCP).

### Ethical consideration

The Health Research Ethics Committee of the Faculty of Medicine Universitas Sumatera Utara approved this study with ethical clearance number 653/KEPK/USU/2025. Permission to access medical records was obtained from Adam Malik General Hospital before data collection. Medical records were accessed for research purposes between 17/07/2025 and 31/12/2025. Authors had access to identifiable participant information during data collection; however, all data were anonymized prior to statistical analysis. Because of the retrospective observational design using secondary medical record data, the requirement for informed consent was waived by the ethics committee.

## Results

### Baseline characteristics of the study population

A total of 606 pediatric outpatients with congenital heart disease fulfilled the eligibility criteria and were included in the final analysis (Table 1). Female patients slightly outnumbered males with 319 cases (52.6%), whereas male patients accounted for 287 cases (47.4%). The largest age category consisted of young toddlers with 166 patients (27.4%), followed by school aged children with 138 patients (22.8%) and infants with 111 patients (18.3%). Preschool children represented the smallest subgroup with 28 patients (4.6%).

**Table 1:** Baseline characteristics of pediatric congenital heart disease outpatients.

Characteristic	Overall cohort (N = 606)
**Sex**	
Female	319 (52.6)
Male	287 (47.4)
**Age group category**	
Adolescent/Neonate	77 (12.7)
Infants	111 (18.3)
Preschool children	28 (4.6)
School aged children	138 (22.8)
Toddlers	86 (14.2)
Young toddlers	166 (27.4)
**Nutritional status**	
Normal	308 (50.8)
Underweight	119 (19.6)
Severe underweight	179 (29.5)
**Healthcare utilization**	
Number of outpatient visits	3.24 ± 2.90
**CHD physiology**	
Non cyanotic CHD	575 (94.9)
Cyanotic CHD	31 (5.1)
**Burden score**	
0	422 (69.6)
1	153 (25.2)
2	26 (4.3)
3	5 (0.8)

Non cyanotic congenital heart disease dominated the cohort and accounted for 575 patients (94.9%), while cyanotic lesions were identified in 31 patients (5.1%). Nutritional assessment demonstrated that 308 patients (50.8%) had normal nutritional status. Underweight and severe underweight status were identified in 119 patients (19.6%) and 179 patients (29.5%), respectively. Overall, malnutrition affected 298 patients (49.2%) within the study population.

The mean number of outpatient visits during 2024 was 3.24 ± 2.90 visits per patient. Most patients demonstrated low cumulative clinical burden scores. A burden score of 0 was identified in 422 patients (69.6%), whereas only five patients (0.8%) demonstrated the highest observed burden score of 3. This distribution was highly skewed toward the lowest score, indicating limited variation in burden across much of the cohort.

### Distribution of congenital heart disease subtypes and nutritional status

Ventricular septal defect represented the most frequently documented congenital heart disease subtype and was identified in 259 patients (33.5%). Patent ductus arteriosus and atrial septal defect followed closely with 218 cases (28.2%) and 217 cases (28.0%), respectively. Pulmonary stenosis accounted for 44 patients (5.7%). Cyanotic lesions occurred less frequently and included transposition of the great arteries in 18 patients (2.3%) and tetralogy of Fallot in 13 patients (1.7%). Coarctation of the aorta represented the rarest lesion with only five documented cases (0.7%) (Table 2).

**Table 2:** Distribution of congenital heart disease subtypes.

CHD subtype	n (%)
Ventricular septal defect (VSD)	259 (33.5)
Patent ductus arteriosus (PDA)	218 (28.2)
Atrial septal defect (ASD)	217 (28.0)
Pulmonary stenosis (PS)	44 (5.7)
Transposition of the great arteries (TGA)	18 (2.3)
Tetralogy of Fallot (TOF)	13 (1.7)
Coarctation of the aorta (CoA)	5 (0.7)

Abbreviations: ASD, atrial septal defect; CoA, coarctation of the aorta; PDA, patent ductus arteriosus; PS, pulmonary stenosis; TGA, transposition of the great arteries; TOF, tetralogy of Fallot; VSD, ventricular septal defect.

Nutritional impairment appeared across both cyanotic and non cyanotic congenital heart disease groups. More than half of patients with tetralogy of Fallot and transposition of the great arteries demonstrated underweight or severe underweight status. Among non cyanotic lesions, ventricular septal defect showed the highest nutritional burden, with severe underweight and underweight collectively affecting more than half of patients in this subgroup. Patients with atrial septal defect and patent ductus arteriosus more frequently demonstrated normal nutritional status compared with other lesions. Coarctation of the aorta showed the most severe nutritional profile, although the subgroup size was very small.

These subtype patterns were interpreted descriptively because the cyanotic subgroup was small (n = 31). Formal inferential comparison between CHD subtypes was therefore limited by sparse subgroup sizes and should be interpreted cautiously.

### Clinical manifestations, complications, and data completeness

Clinical symptoms and complications varied across the cohort (Table 3). Pulmonary hypertension emerged as the most frequently documented complication and affected 115 patients (19.0%). Respiratory tract infection occurred in 56 patients (9.2%), while heart failure was identified in 46 patients (7.6%). Eisenmenger syndrome remained uncommon and was observed in only three patients (0.5%).

**Table 3:** Distribution of clinical complications among pediatric congenital heart disease outpatients.

Complication	n (%)
Pulmonary hypertension	115 (19.0)
Respiratory tract infection	56 (9.2)
Heart failure	46 (7.6)
Eisenmenger syndrome	3 (0.5)

Growth related manifestations also appeared frequently within the outpatient population. Poor weight gain, feeding difficulty, dyspnea, and easy fatigability were commonly documented among patients with repeated healthcare utilization and higher clinical burden scores.

Symptom and complication variables showed substantial missingness because retrospective clinical documentation varied between visits and treating physicians. Detailed completeness assessment and proportions of missing data across study variables are summarized in S1 Table. Because of this missingness, complete case analysis was applied for regression modeling.

### Healthcare utilization patterns

Outpatient healthcare utilization demonstrated substantial variability throughout the study period. The cumulative number of outpatient visits reached 2,065 visits during 2024. Monthly visit frequency ranged from 137 visits in April to 201 visits in July. Higher outpatient volume also appeared during October and November with 198 and 195 visits, respectively.

Evaluation of registration frequency per patient demonstrated that most patients attended outpatient services only once during the study year. Single visit registration was identified in 238 patients (39.3%). Two visits and three visits occurred in 83 patients (13.7%) and 86 patients (14.2%), respectively. A smaller subgroup demonstrated substantially higher healthcare utilization, with several patients requiring more than 10 outpatient visits within one year. The maximum recorded frequency reached 20 visits.

Patients with severe underweight status demonstrated broader healthcare utilization distributions and higher outpatient visit frequencies compared with patients who had normal nutritional status. Violin boxplot analysis also demonstrated wider variability of visit burden among malnourished patients. Detailed healthcare utilization patterns according to nutritional status are illustrated in Fig 1.

**Fig 1:**
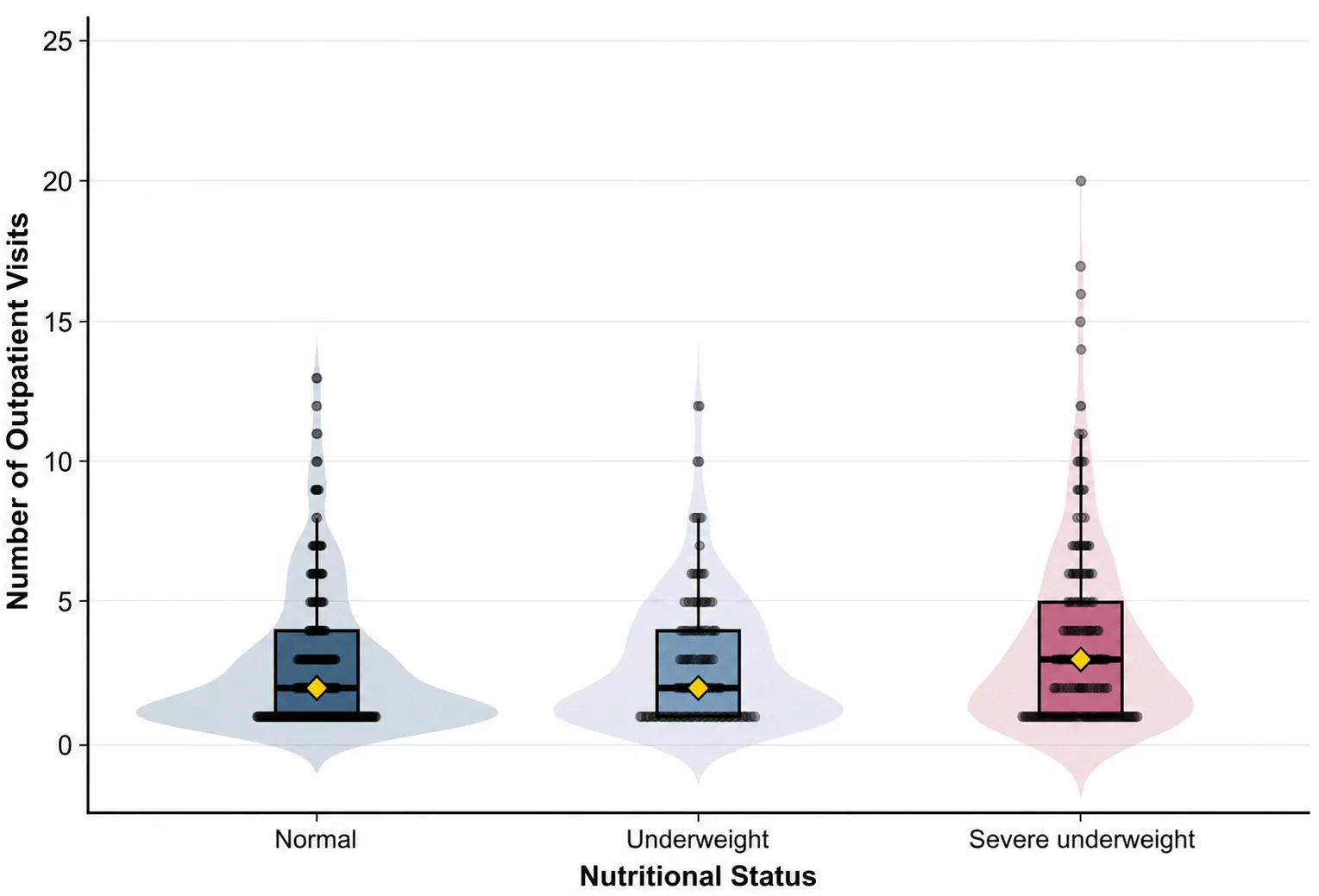
Healthcare utilization across nutritional status groups among pediatric congenital heart disease outpatients. Violin boxplot distribution demonstrating outpatient healthcare utilization according to nutritional status among pediatric patients with congenital heart disease. The violin component represents distribution density, whereas embedded boxplots display the median and interquartile range. Individual observations are overlaid as jittered points, and yellow diamonds indicate group medians.

### Nutritional status across congenital heart disease subtypes

Nutritional impairment appeared across both cyanotic and non cyanotic congenital heart disease groups. More than half of patients with tetralogy of Fallot and transposition of the great arteries demonstrated underweight or severe underweight status. Among non cyanotic lesions, ventricular septal defect showed the highest nutritional burden. Severe underweight and underweight collectively affected more than half of patients with ventricular septal defect.

Patients with atrial septal defect and patent ductus arteriosus more frequently demonstrated normal nutritional status compared with other lesions. Nevertheless, substantial proportions of nutritional impairment still remained evident within these groups. Coarctation of the aorta demonstrated the most severe nutritional profile because all affected patients exhibited malnutrition, although the subgroup size remained very small.

### Factors associated with malnutrition

Univariable logistic regression analysis demonstrated that high healthcare utilization, pulmonary hypertension, poor feeding, and higher burden score were associated with increased odds of malnutrition. Poor feeding demonstrated the strongest crude association and produced nearly fourfold higher odds of malnutrition compared with patients without feeding difficulties. Detailed univariable logistic regression findings are presented in S2 Table.

Multivariable logistic regression analysis demonstrated that younger pediatric age groups exhibited consistently higher odds of malnutrition compared with the adolescent and neonatal reference category (Table 4). Young toddlers demonstrated approximately 2.7-fold increased odds of malnutrition with an adjusted odds ratio of 2.68 (95% CI 1.52–4.74; p < 0.001). Toddlers also demonstrated significantly increased odds with an adjusted odds ratio of 2.28 (95% CI 1.21–4.34; p = 0.012). School aged children and infants showed elevated odds ratios, although confidence intervals crossed the null value. The age distribution of nutritional status is illustrated in Fig 2, which demonstrates a greater proportion of undernutrition among younger pediatric age groups than among older children.

**Table 4:** Multivariable logistic regression analysis of factors associated with malnutrition.

Variable	Adjusted OR	95% CI	p-value
Cyanotic CHD	1.50	0.70–3.20	0.296
Male sex	1.10	0.79–1.52	0.579
**Age group**			
Infants	1.66	0.91–3.05	0.103
Preschool children	0.83	0.33–2.11	0.694
School aged children	1.74	0.98–3.12	0.062
Toddlers	2.28	1.21–4.34	0.012
Young toddlers	2.68	1.52–4.74	<0.001
Burden score	1.50	1.14–1.99	0.004

**Fig 2:**
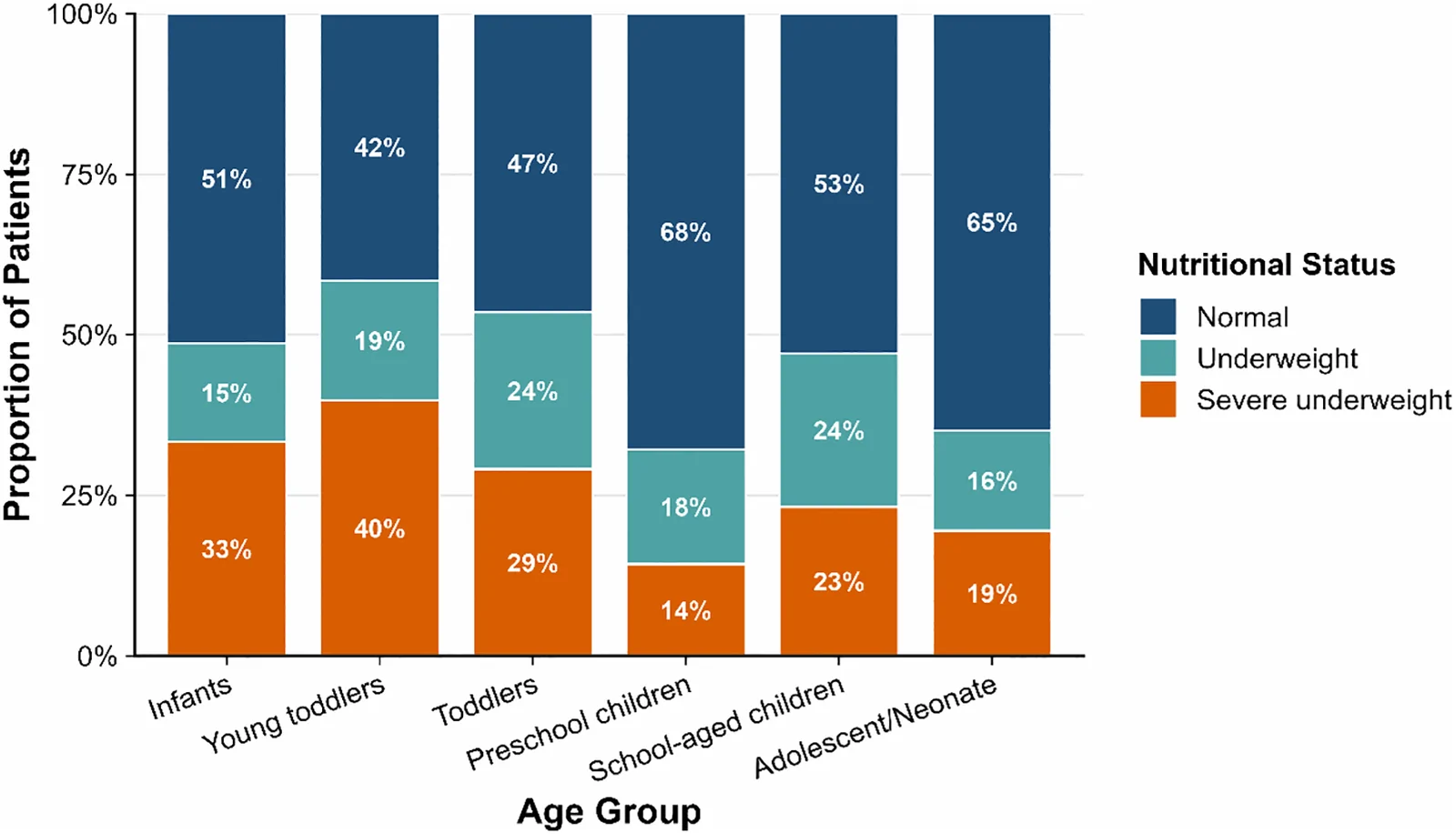
Distribution of nutritional status across pediatric age groups among patients with congenital heart disease. Stacked bars show within age group proportions of normal nutritional status, underweight, and severe underweight. Exact percentages were: infants, 51.4% normal, 15.3% underweight, 33.3% severe underweight; young toddlers, 41.6%, 18.7%, and 39.8%; toddlers, 46.5%, 24.4%, and 29.1%; preschool children, 67.9%, 17.9%, and 14.3%; school aged children, 52.9%, 23.9%, and 23.2%; and adolescent/neonate, 64.9%, 15.6%, and 19.5%.

Higher burden score independently remained associated with malnutrition after adjustment for covariates. Each incremental increase in burden score increased the odds of malnutrition by approximately 50%, with an adjusted odds ratio of 1.50 (95% CI 1.14–1.99; p = 0.004). However, interpretation of this finding should be considered in light of the highly skewed burden score distribution.

Higher burden score independently remained associated with malnutrition after adjustment for covariates. Each incremental increase in burden score increased the odds of malnutrition by approximately 50%, with an adjusted odds ratio of 1.50 (95% CI 1.14–1.99; p = 0.004). However, interpretation of this finding should be considered in light of the highly skewed burden score distribution.

Cyanotic congenital heart disease and male sex did not demonstrate statistically significant associations with malnutrition in the final model. Cyanotic physiology produced an adjusted odds ratio of 1.50 (95% CI 0.70–3.20; p = 0.296), whereas male sex produced an adjusted odds ratio of 1.10 (95% CI 0.79–1.52; p = 0.579). Adjusted odds ratios and confidence intervals derived from the final multivariable logistic regression model are visualized in Fig 3.

**Fig 3:**
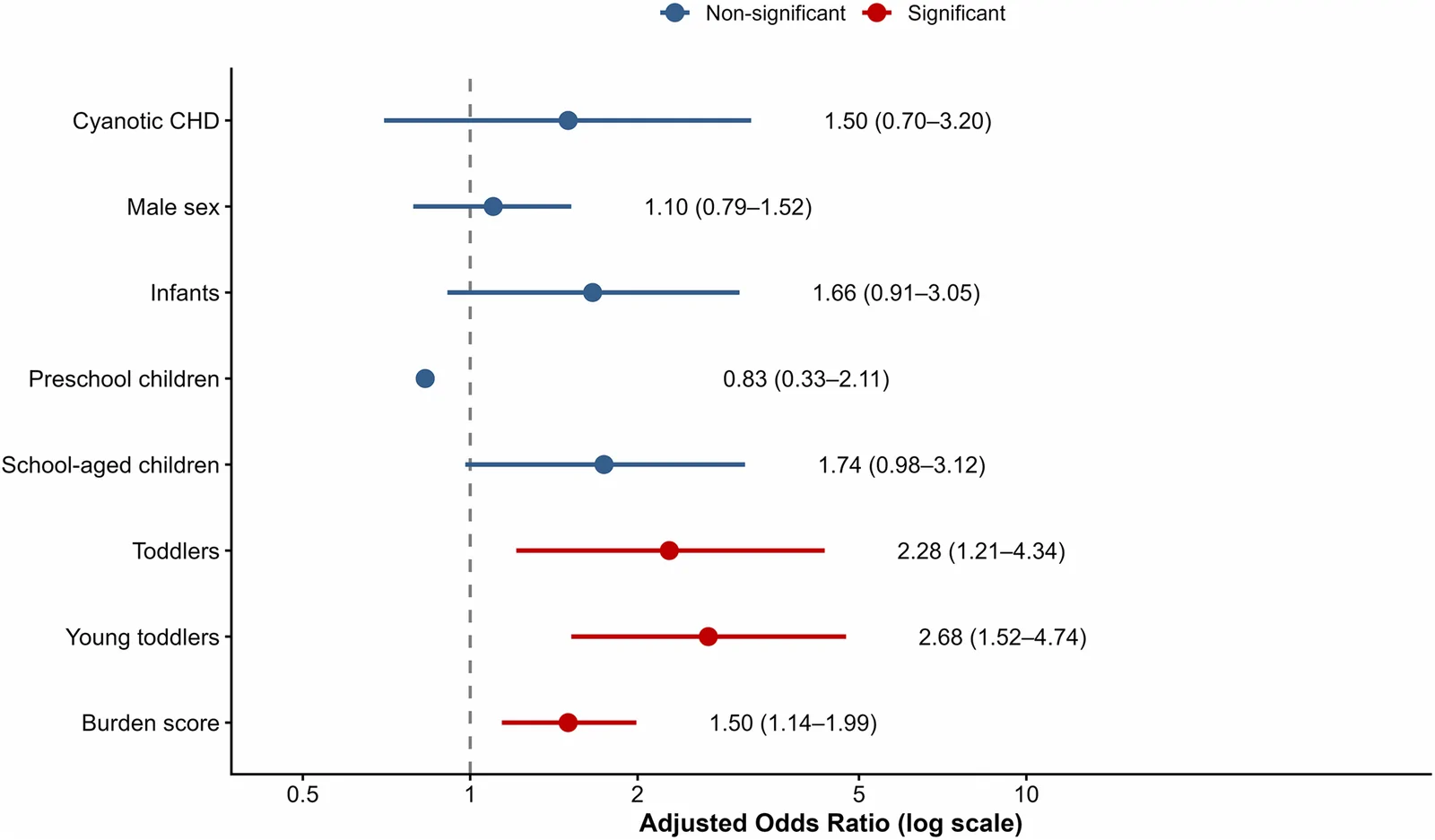
Factors associated with malnutrition among pediatric congenital heart disease outpatients. Forest plot showing adjusted odds ratios and 95% confidence intervals derived from the multivariable logistic regression model evaluating factors associated with malnutrition among pediatric congenital heart disease outpatients.

### Sensitivity analysis and model performance

Sensitivity analysis using the original non collapsed age group classification demonstrated findings generally consistent with the primary model. Young toddlers and toddlers remained significantly associated with increased odds of malnutrition, while high healthcare utilization also remained independently associated with malnutrition in the sensitivity model. High healthcare utilization was associated with greater odds of malnutrition (adjusted OR 1.54, 95% CI 1.07–2.22; p = 0.020). Estimates for the neonatal subgroup remained unstable because of the very small number of observations and quasi complete separation.

Multicollinearity assessment demonstrated acceptable generalized variance inflation factor values across included covariates. No evidence of clinically significant multicollinearity was identified within the final regression model. Detailed multicollinearity diagnostics are presented in S4 Table.

Overall model performance demonstrated acceptable calibration and classification capability for exploratory clinical association analysis. Detailed model performance metrics are summarized in S5 Table. The final multivariable model produced Akaike information criterion 828.9 and Tjur’s R² 0.062. Calibration analysis demonstrated reasonable agreement between predicted and observed probabilities of malnutrition across the range of estimated risk values. Calibration performance of the final multivariable model is illustrated in S1 Fig.

## Discussion

This study demonstrated that non cyanotic congenital heart disease dominated the outpatient pediatric population at Adam Malik General Hospital, with ventricular septal defect, patent ductus arteriosus, and atrial septal defect representing the most frequently identified lesions. Nearly half of all patients experienced malnutrition, while pulmonary hypertension emerged as the most commonly documented complication. Younger pediatric age groups and higher cumulative clinical burden were independently associated with greater odds of malnutrition in the primary multivariable model. Higher healthcare utilization was also associated with malnutrition in univariable analysis and remained statistically significant in the sensitivity model, but this finding should be interpreted separately from the primary multivariable results.

The predominance of ventricular septal defect, patent ductus arteriosus, and atrial septal defect is also consistent with reports from Southeast Asia. Previous Indonesian studies from Medan and Bandung similarly identified acyanotic or left-to-right shunt lesions as the predominant CHD phenotype, with ventricular septal defect representing the most common diagnosis. The Medan cohort reported malnutrition in approximately 37% of children with CHD, whereas our study observed malnutrition in nearly half of the outpatient population, suggesting a greater nutritional burden in our cohort. A study from Bandung likewise demonstrated that malnutrition was associated with recurrent pneumonia among children with left-to-right shunt CHD, reinforcing the close interaction between nutritional impairment and chronic clinical morbidity in Indonesian children with congenital heart disease [9–12].

Young toddlers represented the largest age group within the present cohort. This distribution likely reflects the period when persistent symptoms, growth disturbances, recurrent respiratory infections, or delayed developmental progress become more apparent to caregivers and healthcare providers. Children within this age range also undergo rapid somatic growth and increased nutritional demands, making underlying hemodynamic abnormalities more clinically visible. School aged children formed the second largest subgroup and may reflect long term outpatient follow up among survivors with stable congenital lesions requiring periodic cardiac evaluation. Earlier studies from tertiary referral centers have reported comparable age distributions with continued healthcare utilization extending beyond infancy [11,13].

Nearly half of the study population demonstrated underweight or severe underweight nutritional status. This finding reinforces the substantial nutritional burden carried by children with congenital heart disease in resource limited settings. Chronic hemodynamic stress may be associated with increased resting energy expenditure and impaired feeding patterns. Pulmonary overcirculation, recurrent respiratory symptoms, and heart failure further increase metabolic demand while reducing caloric intake efficiency. Recurrent illness and prolonged inflammatory stress may also be associated with impaired nutrient absorption and weight gain during critical periods of childhood growth [3,4,8,14].

Nutritional impairment appeared across both cyanotic and non cyanotic lesions. Although cyanotic congenital heart disease has traditionally been associated with severe malnutrition because of chronic hypoxemia, ventricular septal defect demonstrated similarly high rates of nutritional problems within this cohort. Large left to right shunts may be associated with pulmonary congestion, tachypnea, feeding intolerance, and recurrent respiratory tract infections, which may contribute to impaired growth despite preserved systemic oxygenation. This observation suggests that chronic hemodynamic burden may contribute to malnutrition as strongly as cyanotic physiology itself [15,16].

Similar observations have been reported in Thailand, where approximately two – thirds of children undergoing corrective cardiac surgery were malnourished before intervention. However, unlike the Thai surgical cohort, cyanotic physiology was not independently associated with malnutrition in our study. This discrepancy likely reflects differences in study populations, as the Thai study enrolled children awaiting surgery, whereas the present investigation evaluated a heterogeneous outpatient population with varying disease severity and follow – up duration. These findings suggest that cumulative clinical burden and persistent hemodynamic stress may have a stronger association with nutritional status than cyanotic physiology alone in this outpatient population [17].

Pulmonary hypertension represented the most common complication identified in the study population. Persistent exposure to increased pulmonary blood flow in untreated or hemodynamically significant shunt lesions may be related to this finding. Pulmonary vascular remodeling may develop progressively when left to right shunting remains uncontrolled over prolonged periods. Previous studies have also documented pulmonary hypertension as a frequent complication among pediatric patients with congenital heart disease, especially in settings where delayed diagnosis or limited access to definitive intervention remains common [3,6,14].

Healthcare utilization patterns demonstrated wide variability across the cohort. Most patients attended outpatient services only once or twice during the study year, whereas a smaller subgroup required repeated follow up with substantially higher visit frequencies. Patients with severe underweight status showed broader healthcare utilization distributions compared with children who maintained normal nutritional status. In univariable analysis, high healthcare utilization was associated with greater odds of malnutrition, and this association remained statistically significant in the sensitivity model (adjusted OR 1.54, 95% CI 1.07–2.22; p = 0.020). However, because healthcare utilization was not included in the primary multivariable model, this finding should not be interpreted as an independent association in the primary analysis. The observed relationship may reflect greater clinical burden or other factors that were not fully captured in the retrospective medical records [9,12].

Multivariable regression analysis identified young toddlers and toddlers as the age groups with the highest odds of malnutrition. Early childhood represents a vulnerable developmental period characterized by rapid growth velocity and increased nutritional demand. Children with congenital heart disease may have difficulty meeting these metabolic requirements because chronic cardiac dysfunction may be associated with reduced energy utilization and feeding capacity. Changes in dietary intake during early childhood may also be associated with nutritional vulnerability particularly among children with persistent respiratory symptoms or feeding difficulties [4,8,14].

Sensitivity analysis using the original age group classification produced findings that were generally consistent with the primary analysis, although estimates for the neonatal subgroup remained unstable because of the very small number of observations (S3 Table).

Higher burden scores were associated with greater odds of malnutrition after adjustment for the prespecified covariates. This finding is consistent with the concept that nutritional deterioration in congenital heart disease reflects cumulative clinical stress rather than a single isolated manifestation. Recurrent respiratory symptoms, feeding problems, pulmonary hypertension, and repeated healthcare encounters may collectively reflect greater clinical complexity and nutritional vulnerability. Burden based assessment may therefore provide a broader representation of disease complexity among pediatric congenital heart disease outpatients [10,12,18].

Cyanotic physiology did not demonstrate a statistically significant association with malnutrition after adjustment for covariates. Several explanations may account for this finding. First, the relatively small number of cyanotic cases reduced statistical power for subgroup comparison. Second, non cyanotic lesions with substantial left to right shunting may produce severe metabolic stress and feeding dysfunction comparable to cyanotic lesions. Third, outpatient survivors with cyanotic congenital heart disease may already represent a relatively stable subgroup receiving long term specialized care. Similar observations have been reported by studies demonstrating that nutritional impairment in congenital heart disease depends not only on oxygen saturation status but also on hemodynamic severity and chronic symptom burden.

The present study has several limitations. The retrospective design relied on medical record documentation that demonstrated substantial missingness within symptom and complication variables. Detailed completeness assessment and proportions of missing data across study variables are summarized in S1 Table. The cumulative burden score was an investigator developed exploratory measure rather than a validated clinical instrument; therefore, its findings should be interpreted cautiously and require external validation. The analysis classified congenital heart disease according to cyanotic and non-cyanotic physiology because detailed lesion hemodynamic severity parameters (echocardiographic measures of shunt magnitude, ventricular function, and pressure gradients) were not consistently available in the medical records and therefore could not be included in the multivariable analyses. Residual confounding by disease severity cannot be excluded and should be considered when interpreting the observed associations. In addition, the study was conducted at a single tertiary referral center, which introduces referral bias. Adam Malik General Hospital receives patients from multiple districts across North Sumatra and neighboring provinces, resulting in a cohort enriched with children requiring specialized cardiac evaluation, long term follow – up, or management of more complex disease. Therefore, these findings should be interpreted as representative of a tertiary care outpatient population rather than the overall pediatric CHD population in Indonesia.

The findings support routine nutritional screening among children with congenital heart disease, particularly younger children and those with greater cumulative clinical burden. These patients may warrant closer nutritional assessment and multidisciplinary follow-up as part of ongoing outpatient care.

## Conclusion

Pediatric outpatients with congenital heart disease at Adam Malik General Hospital had a substantial burden of malnutrition, with nearly half of the cohort classified as underweight or severely underweight. Younger age groups and higher cumulative clinical burden were associated with greater odds of malnutrition in the primary multivariable model. Healthcare utilization was associated with malnutrition in univariable analysis and remained statistically significant in the sensitivity analysis, but it was not retained in the primary multivariable model. These findings should be interpreted cautiously because the retrospective cross-sectional design does not establish temporal or causal relationships while missing clinical data and unmeasured disease severity may have influenced the observed associations. In practice, routine nutritional screening during pediatric CHD follow-up, with particular attention to younger children and those with greater documented clinical burden, may facilitate earlier nutritional assessment and appropriate multidisciplinary management. Prospective studies using standardized nutritional and clinical severity measures are needed to confirm these associations.

## Supporting information

S1 Table Completeness assessment of variables included in the retrospective pediatric congenital heart disease cohort. Abbreviations: CHD, congenital heart disease. Data completeness was evaluated across all study variables included in the retrospective outpatient cohort. Symptom- and complication-related variables demonstrated substantial missingness because of inconsistent retrospective clinical documentation. No imputation procedures were performed, and complete-case analysis was applied for multivariable regression modeling.
(DOCX)

S2 Table Univariable logistic regression analysis of factors associated with malnutrition among pediatric congenital heart disease outpatients. Odds ratios (ORs) greater than 1 indicate increased odds of malnutrition.
(DOCX)

S3 Table Sensitivity analysis using the original age-group classification in multivariable logistic regression. Sensitivity analysis was performed using the original age-group classification to evaluate the robustness of the primary multivariable logistic regression findings. Adjusted odds ratios (ORs) with 95% confidence intervals (CIs) were derived from logistic regression models with malnutrition as the dependent variable. Reference categories were non-cyanotic CHD, female sex, and preschool-aged children. Odds ratios greater than 1 indicate increased odds of malnutrition. NE = not estimable with reliable precision because of sparse observations and quasi-complete separation in the neonatal subgroup (n = 4). Despite instability in this subgroup, the overall direction and magnitude of associations remained consistent with the primary analysis, supporting the robustness of the primary findings.
(DOCX)

S4 Table Multicollinearity diagnostics for the multivariable logistic regression model. GVIF = generalized variance inflation factor. Adjusted GVIF was calculated as GVIF^(1/(2×Df))^ to account for variables with multiple degrees of freedom. Higher adjusted GVIF values indicate greater collinearity among predictors. All adjusted GVIF values remained below 5, suggesting no evidence of clinically significant multicollinearity within the final multivariable regression model. Df denotes degrees of freedom for each variable.
(DOCX)

S5 Table Overall performance metrics of the multivariable logistic regression model. Model performance metrics were calculated to evaluate overall goodness-of-fit, explanatory performance, and classification accuracy of the final multivariable logistic regression model. Lower AIC, AICc, BIC, RMSE, and log-loss values indicate improved model fit. Tjur’s R^2^ reflects explanatory performance for binary outcomes, whereas PCP represents the proportion of correctly classified observations. Collectively, these metrics suggested acceptable overall model performance for exploratory clinical association analysis.
(DOCX)

S1 Fig Calibration plot of the final multivariable logistic regression model for malnutrition among pediatric congenital heart disease outpatients. Calibration plot demonstrating agreement between predicted and observed probabilities of malnutrition derived from the multivariable logistic regression model. The dashed diagonal line represents perfect calibration, indicating exact agreement between predicted and observed risk estimates. Blue points and the solid calibration curve represent bootstrap-corrected calibration estimates generated using 1,000 bootstrap resamples. Overall, the model demonstrated acceptable calibration across the observed range of predicted probabilities, although minor deviation from perfect calibration was observed at higher predicted probabilities.
(TIF)

## References

[1] Liu Y, Chen S, Zühlke L, Black GC, Choy MK, Li N, et al. Global birth prevalence of congenital heart defects 1970–2017: Updated systematic review and meta-analysis of 260 studies. Int J Epidemiol. 2019;48(2):455–63. doi: 10.1093/ije/dyz009 30783674 PMC6469300

[2] Zhao Q-M, Liu F, Wu L, Ma X-J, Niu C, Huang G-Y. Prevalence of congenital heart disease at live birth in China. J Pediatr. 2019;204:53–8. doi: 10.1016/j.jpeds.2018.08.040 30270157

[3] Lee J, Marshall T, Buck H, Pamela M, Daack-Hirsch S. Growth failure in children with congenital heart disease. Children (Basel). 2025;12(5):616. doi: 10.3390/children12050616 40426795 PMC12110605

[4] Jamei Khosroshahi A, Shoaran M, Ghaffari S, Shabanpour E, Seraj Ebrahimi P, Ansari A, et al. Growth pattern of children with congenital heart disease before and after open heart surgery. Front Pediatr. 2025;13:1463998. doi: 10.3389/fped.2025.1463998 40777158 PMC12328431

[5] Jung SY. Growth in children with congenital heart disease: Filling the gap with nationwide evidence. Korean Circ J. 2025;55(10):936–7. doi: 10.4070/kcj.2025.0139 40537436 PMC12599446

[6] Cha JH, Choi Y-J, Ryu S, Cho Y, Yang S, Na JY. Physical growth trajectories in children with congenital heart disease: A nationwide study. Korean Circ J. 2025;55(10):923–35. doi: 10.4070/kcj.2025.0020 40537424 PMC12599410

[7] Benderly M, Buber J, Kalter-Leibovici O, Blieden L, Dadashev A, Lorber A, et al. Israeli Adult Congenital Heart Disease Research Group. Health service utilization patterns among adults with congenital heart disease: a population-based study. J Am Heart Assoc. 2021;10(2):e018037. doi: 10.1161/JAHA.120.018037 33432841 PMC7955316

[8] Abbas Q, Ali H, Ahuja AK, Bhatti OA, Ladak S, Khan I, et al. Preoperative nutrition status in children with congenital heart disease and its impact on postoperative outcomes: A systematic review and meta-analysis. Sci Rep. 2025;15(1):25738. doi: 10.1038/s41598-025-96374-z 40670722 PMC12267774

[9] Amelia P, Yosephine AG, Tobing TC, Savira M, Viandy V, Inglin M. Association between type of congenital heart disease with child growth and development status: A cross-sectional study in Medan, Indonesia. Narra J. 2023;3(3):e414. doi: 10.52225/narra.v3i3.414 38450335 PMC10915998

[10] Rahayuningsih SE, Budi Kuswiyanto R, Reviyani Suryaningrat F, Nataprawira HM, Sukadi A. Left to right shunt congenital heart disease as a risk factor of recurrent pneumonia in under five-year-old children: A single centre experience in Bandung Indonesia. Med Glas (Zenica). 2021;18(1):33–7. doi: 10.17392/1196-21 33155460

[11] Thomford NE, Biney RP, Okai E. Clinical spectrum of congenital heart defects detected at the child health clinic in a tertiary health facility in Ghana: A retrospective analysis. J Congenit Heart Dis. 2020;4:3. doi: 10.1186/s40949-020-00034-y

[12] Finariawan F, Mahmud SA. The characteristics and distribution of congenital heart disease in outpatient clinic and inpatient ward of RSUD Dr. Soedono Madiun East Java in year 2015. Acta Cardiol Indones. 2018;4(1):9. doi: 10.22146/aci.36633

[13] Baksh AK, Waworuntu DS, Umboh A. Profil penyakit jantung bawaan di Bagian Ilmu Kesehatan Anak RSUP Prof. Dr. R. D. Kandou Manado periode September 2022–Agustus 2023. Acta Cardiol Indones. 2023;12(3):55352. doi: 10.35790/ecl.v12i3.55352

[14] Woldesenbet R, Murugan R, Mulugeta F, Moges T. Nutritional status and associated factors among children with congenital heart disease in selected governmental hospitals and cardiac center, Addis Ababa Ethiopia. BMC Pediatr. 2021;21(1):538. doi: 10.1186/s12887-021-03023-1 34856935 PMC8638350

[15] Jone P-N, Ivy DD, Hauck A, Karamlou T, Truong U, Coleman RD, et al. Pulmonary hypertension in congenital heart disease: A scientific statement from the American Heart Association. Circ Heart Fail. 2023;16(7):e00080. doi: 10.1161/HHF.0000000000000080 37357777

[16] Loni R, Ranganath P, Juvekar M, Tikare N, Bidari LH, Karva MM. Clinical profile and outcome of children with congenital heart diseases admitted with acute events in a paediatric tertiary care unit in North Karnataka. Int J Contemp Pediatr. 2021;8(8). doi: 10.18203/2349-3291.ijcp20212757

[17] Sethasathien S, Silvilairat S, Sittiwangkul R, Makonkawkeyoon K, Kittisakmontri K, Pongprot Y. Prevalence and predictive factors of malnutrition in Thai children with congenital heart disease and short-term postoperative growth outcomes. Nutr Health. 2023;29(3):549–55. doi: 10.1177/02601060221082382 35238234

[18] Kempny A, Diller G-P, Dimopoulos K, Alonso-Gonzalez R, Uebing A, Li W, et al. Determinants of outpatient clinic attendance amongst adults with congenital heart disease and outcome. Int J Cardiol. 2016;203:245–50. doi: 10.1016/j.ijcard.2015.10.081 26519677

