# Supplementary material for: Nutritional status, clinical burden, and healthcare utilization among pediatric outpatients with congenital heart disease: A retrospective cross-sectional study from Indonesia
Source: PLoS One. 2026 Oct 8;21(10):e0351014. doi: 10.1371/journal.pone.0351014 (PMC13648971; doi:10.1371/journal.pone.0351014)
Supplement: Supplementary file 2 [file pone.0351014.s001.docx]

Congenital Heart Disease Cohort

| **Domain** | **Variable** | **Missing observations, n** | **Missingness (%)** | **Data completeness** |
| --- | --- | --- | --- | --- |
| Patient identifiers | Medical record number | 0 | 0.0 | Complete |
| Patient identifiers | Patient name | 0 | 0.0 | Complete |
| Demographics | Sex | 0 | 0.0 | Complete |
| Demographics | Age group | 0 | 0.0 | Complete |
| Clinical characteristics | CHD subtype | 0 | 0.0 | Complete |
| Nutritional assessment | Nutritional status | 0 | 0.0 | Complete |
| Clinical presentation | Symptoms | 318 | 52.5 | Substantial missingness |
| Clinical presentation | Complications | 384 | 63.4 | Substantial missingness |
| Healthcare utilization | Number of outpatient visits | 0 | 0.0 | Complete |

Abbreviations: CHD, congenital heart disease.

Data completeness was evaluated across all study variables included in the retrospective outpatient cohort. Symptom and complication related variables demonstrated substantial missingness because of inconsistent retrospective clinical documentation. However, these variables were not included in the final multivariable logistic regression model. The final regression model included cyanotic CHD, sex, age-group category, and burden score, all of which had complete data for all 606 patients. Consequently, all variables included in the final regression model were complete; therefore all 606 patients were retained and no observations were excluded.
