# Supplementary material for: Nutritional status, clinical burden, and healthcare utilization among pediatric outpatients with congenital heart disease: A retrospective cross-sectional study from Indonesia
Source: PLoS One. 2026 Oct 8;21(10):e0351014. doi: 10.1371/journal.pone.0351014 (PMC13648971; doi:10.1371/journal.pone.0351014)
Supplement: Supplementary file 3 [file pone.0351014.s002.docx]

S2 Table. Univariable Logistic Regression Analysis of Factors Associated With Malnutrition

| **Variable** | **Crude OR** | **95% CI** | **p-value** |
| --- | --- | --- | --- |
| Cyanotic CHD | 1.46 | 0.71–3.10 | 0.312 |
| Male sex | 1.14 | 0.83–1.57 | 0.428 |
| High healthcare utilization | 1.58 | 1.12–2.22 | 0.009 |
| Pulmonary hypertension | 1.64 | 1.09–2.49 | 0.018 |
| Heart failure | 1.14 | 0.62–2.09 | 0.672 |
| Dyspnea | 1.20 | 0.81–1.79 | 0.362 |
| Poor feeding | 3.75 | 1.33–13.34 | 0.021 |
| Burden score | 1.44 | 1.10–1.90 | 0.009 |

Odds ratios (ORs) greater than 1 indicate increased odds of malnutrition.
