# Supplementary material for: Nutritional status, clinical burden, and healthcare utilization among pediatric outpatients with congenital heart disease: A retrospective cross-sectional study from Indonesia
Source: PLoS One. 2026 Oct 8;21(10):e0351014. doi: 10.1371/journal.pone.0351014 (PMC13648971; doi:10.1371/journal.pone.0351014)
Supplement: Supplementary file 4 [file pone.0351014.s003.docx]

S3 Table. Sensitivity Analysis Using the Original Age-Group Classification in Multivariable Logistic Regression

| **Variable** | **Adjusted OR** | **95% CI** | **p-value** |
| --- | --- | --- | --- |
| Cyanotic CHD | 1.58 | 0.74–3.67 | 0.235 |
| Male sex | 1.10 | 0.79–1.54 | 0.558 |
| **Age group** |  |  |  |
| Early adolescents | 1.31 | 0.47–3.49 | 0.608 |
| Infants | 2.24 | 0.93–5.23 | 0.075 |
| Neonates | NE* | NE* | 0.975 |
| Late adolescents | 1.04 | 0.34–3.14 | 0.949 |
| School-aged children | 1.93 | 0.83–4.44 | 0.137 |
| Toddlers | 2.49 | 1.01–6.07 | 0.049 |
| Young toddlers | 2.80 | 1.20–6.44 | 0.019 |
| High healthcare utilization | 1.54 | 1.07–2.22 | 0.020 |

Sensitivity analysis was performed using the original age-group classification to evaluate the robustness of the primary multivariable logistic regression findings. Adjusted odds ratios (ORs) with 95% confidence intervals (CIs) were derived from logistic regression models with malnutrition as the dependent variable. Reference categories were non-cyanotic CHD, female sex, and preschool-aged children. Odds ratios greater than 1 indicate increased odds of malnutrition.

*NE = not estimable with reliable precision because of sparse observations and quasi-complete separation in the neonatal subgroup (n = 4). Despite instability in this subgroup, the overall direction and magnitude of associations remained consistent with the primary analysis, supporting the robustness of the primary findings.
