# Supplementary material for: Nutritional status, clinical burden, and healthcare utilization among pediatric outpatients with congenital heart disease: A retrospective cross-sectional study from Indonesia
Source: PLoS One. 2026 Oct 8;21(10):e0351014. doi: 10.1371/journal.pone.0351014 (PMC13648971; doi:10.1371/journal.pone.0351014)
Supplement: Supplementary file 5 [file pone.0351014.s004.docx]

S4 Table. Multicollinearity Diagnostics for the Multivariable Logistic Regression Model

| **Variable** | **GVIF** | **Df** | **Adjusted GVIF*** |
| --- | --- | --- | --- |
| Cyanotic CHD | 1.03 | 1 | 1.01 |
| Sex | 1.02 | 1 | 1.01 |
| Age-group category | 1.22 | 5 | 1.02 |
| Burden score | 4.59 | 1 | 2.14 |

*GVIF = generalized variance inflation factor. Adjusted GVIF was calculated as GVIF^(1/(2×Df))^ to account for variables with multiple degrees of freedom. Higher values indicate greater collinearity among predictors. Adjusted GVIF values below 5 were considered acceptable and suggested no evidence of clinically significant multicollinearity in the final regression model. Df denotes degrees of freedom for each variable.
