# Supplementary material for: Nutritional status, clinical burden, and healthcare utilization among pediatric outpatients with congenital heart disease: A retrospective cross-sectional study from Indonesia
Source: PLoS One. 2026 Oct 8;21(10):e0351014. doi: 10.1371/journal.pone.0351014 (PMC13648971; doi:10.1371/journal.pone.0351014)
Supplement: Supplementary file 6 [file pone.0351014.s005.docx]

S5 Table. Overall Performance Metrics of the Multivariable Logistic Regression Model

| **Metric** | **Value** |
| --- | --- |
| Akaike Information Criterion (AIC) | 828.9 |
| Corrected AIC (AICc) | 829.6 |
| Bayesian Information Criterion (BIC) | 890.6 |
| Tjur’s R² | 0.062 |
| Root Mean Square Error (RMSE) | 0.484 |
| Log-loss | 0.661 |
| Proportion Correctly Predicted (PCP) | 0.531 |

Model performance metrics were calculated to evaluate overall goodness-of-fit, explanatory performance, and classification accuracy of the final multivariable logistic regression model. Lower AIC, AICc, BIC, RMSE, and log-loss values indicate improved model fit. Tjur’s R² reflects explanatory performance for binary outcomes, whereas PCP represents the proportion of correctly classified observations. Collectively, these metrics suggested acceptable overall model performance for exploratory clinical association analysis.
