# Supplementary material for: Nutritional status, clinical burden, and healthcare utilization among pediatric outpatients with congenital heart disease: A retrospective cross-sectional study from Indonesia
Source: PLoS One. 2026 Oct 8;21(10):e0351014. doi: 10.1371/journal.pone.0351014 (PMC13648971; doi:10.1371/journal.pone.0351014)
Supplement: Supplementary file 8 [file pone.0351014.s009.docx]

Comments

This manuscript addresses an important and clinically relevant topic by evaluating nutritional status, clinical burden, and healthcare utilization among pediatric patients with congenital heart disease in a tertiary referral hospital in Indonesia. The study provides valuable information from a resource-limited setting, where evidence regarding nutritional challenges among children with congenital heart disease remains limited. The relatively large sample size, integration of nutritional and clinical characteristics, and use of multivariable analysis are notable strengths.

However, several aspects require clarification and improvement to strengthen the manuscript.

**Major comments:**

1. Statistical analysis and reporting

The statistical methods appear generally appropriate; however, further details are needed to evaluate the rigor of the analysis. The authors should clarify the criteria used for selecting variables included in the multivariable logistic regression model, the number of participants included in the final regression analysis after handling missing data, and whether potential confounding factors were considered. Reporting adjusted odds ratios with 95% confidence intervals in the abstract would improve transparency.

2. Definition of cumulative clinical burden score

The cumulative clinical burden score is an important variable in this study; however, its development requires further explanation. The authors should clarify the rationale for selecting included symptoms and complications, whether all components were equally weighted, and whether the score was assessed for reliability or validity.

3. Missing data management

The manuscript reports substantial missingness for some clinical variables and applies complete-case analysis. The authors should provide more detailed information regarding the proportion of missing data for each variable and discuss the potential risk of selection bias resulting from excluding cases with incomplete information.

4. Interpretation of healthcare utilization findings

Healthcare utilization is an important component of the study; however, the analysis remains mainly descriptive. The authors should consider providing statistical comparisons between nutritional groups or clarify that healthcare utilization patterns are descriptive findings rather than identified predictors.

5. Causal interpretation

Given the retrospective cross-sectional design, causal relationships between malnutrition, clinical burden, and healthcare utilization cannot be established. The authors should ensure that the wording throughout the manuscript reflects associations rather than causal effects.

**Minor comments**:

Include prevalence estimates and adjusted odds ratios with 95% confidence intervals in the abstract. This would substantially improve its scientific value and compliance with reporting standards.

The abstract would be strengthened by including key quantitative regression results, including adjusted odds ratios, confidence intervals, and p-values.

Please ensure consistent terminology throughout the manuscript. For example:

Use “acyanotic congenital heart disease” rather than “non-cyanotic congenital heart disease.”

Use “resource-limited settings” rather than “resource limited settings.”

Some sentences, particularly in the Introduction and Discussion, are lengthy and could be revised for improved readability.

The Discussion contains some repetition regarding the relationship between malnutrition and disease burden. These sections could be condensed to improve focus.

The limitations section is appropriate but could further discuss the implications of single-center data and referral-center bias.

**Overall assessment:**

The manuscript presents important findings and has potential value for pediatric cardiology and global health research. The study would benefit from further methodological clarification, improved statistical reporting, and minor language refinement before it can be considered for publication. **I recommend major revision.**
