# Supplementary material for: Nutritional status, clinical burden, and healthcare utilization among pediatric outpatients with congenital heart disease: A retrospective cross-sectional study from Indonesia
Source: PLoS One. 2026 Oct 8;21(10):e0351014. doi: 10.1371/journal.pone.0351014 (PMC13648971; doi:10.1371/journal.pone.0351014)
Supplement: Supplementary file 10008 [file pone.0351014.s008.pdf]

ScreenIT Report

This is an automated report for 10.64898+2026.05.23.26353925.pdf. This report was solicited by the PLOS One editorial team and provided by ScreenIT.

ScreenIT is an independent group of scientists developing automated tools that analyze academic papers. A set of automated tools screened your submitted manuscript and provided the report below. Each tool was created by your academic colleagues with the goal of helping authors.

The tools look for factors that are important for transparency, rigor and reproducibility, and we hope that the report might help you to improve reporting in your manuscript. Within the report you will find links to more information about the items that the tools check. These links include helpful papers, websites, or videos that explain why the item is important.

While our screening tools aim to improve and maintain quality standards they may, on occasion, miss nuances specific to your study type or flag something incorrectly. Each tool has limitations that are described on the ScreenIT website. The tools screen the main file for the paper; they are not able to screen supplements stored in separate files.

Please note that the Academic Editor had access to these comments while making a decision on your manuscript. The Academic Editor may ask that issues flagged in this report be addressed.

If you would like to provide feedback on the ScreenIT tool, please email the team at ScreenIt@bih-charite.de. If you have questions or concerns about the review process, please contact the PLOS One office at plosone@plos.org.

How to read the report:

- no action required

? - review for potential issues

! - a critical issue was found by a tool

n/a - may not be applicable to your paper

- more information about this item can be obtained using this link

Rigor

	Ethics	IRB: "67 Ethical consideration The Health Research Ethics Committee of the Faculty of Medicine Universitas Sumatera Utara approved this study with ethical clearance number 653/ KEPK/USU/2025."<br>Field Sample Permit: "Permission to access medical records was obtained from Adam Malik General Hospital before data collection."
	Inclusion and Exclusion Criteria	"Inclusion criteria consisted of pediatric patients aged 0 to 18 years with a confirmed diagnosis of congenital heart disease documented through echocardiographic evaluation in the medical record."
	Flow charts and attrition	We did not find a study flow chart of excluded observations. We strongly recommend using flow charts because they provide an overview of the study design and more information about attrition. If you included a study flow chart in your supplemental files, we apologize for missing this. Our tool is not able to screen separate supplemental files.<br><br>Sentence about attrition: not detected. Please provide information about the drop-out of subjects, or loss of animals or samples. This could be done using a study flow chart, or described in the text.
	Sex as a biological variable	"Female patients slightly outnumbered males with 319 cases (52.6%), whereas male patients accounted for 287 cases (47.4%)."
	Subject Demographics	Age: "Inclusion criteria consisted of pediatric patients aged 0 to 18 years with a confirmed diagnosis of congenital heart disease documented through echocardiographic evaluation in the medical record."
	Randomization	"No imputation procedures were performed because of the retrospective nature of the dataset and the non random pattern of missingness."
	Blinding	Not detected. Please specify whether blinding was used at various phases of the

?		experiment (e.g., blinding of patients, caregivers, outcome assessments, data analysis).
?	Power Analysis	Not detected. Please state how you determined the sample size for the study. If you performed a power or sample size calculation, please provide details.
n/a	Replication	not required.

Generated by Barzooka (flow charts), SciScore (everything else).
SciScore only screens the text of the methods section; we apologize if the tool has missed items reported elsewhere in the paper or supplements.

Resources

Persistent unique identifiers for research resources, RRIDs, can be used to track these key resources though the literature. They are requested or required in many journals, but even if your journal does not require them, we strongly encourage the use of these for the readers of papers who wish to easily track down the key materials like antibodies, software tools, or mice.

Software and Algorithms	"146 Statistical analysis Statistical analysis was performed using IBM SPSS Statistics and R statistical software."	? SPSS suggested: SPSS, RRID:SCR_002865. Please make sure this RRID suggestion is accurate before including in your manuscript.

Generated by SciScore.
SciScore only screens the text of the methods section; we apologize if the tool has missed items reported elsewhere in the paper or supplements.

Transparency

?	Open code	If you wrote code to analyze your data, please consider sharing the code in a public repository. This makes it easier for others to reproduce your analyses, and may also aid others seeking to analyze similar datasets.
?	Open data	If permitted, sharing data on a public repository with a persistent identifier (a DOI or accession number) can improve reproducibility

		and make it easier for other scientists to expand on your work. Papers with open data are cited more often than papers without open data. Some institutions have an expert who can provide advice on data sharing.
✓	Self-acknowledged limitations	ⓘ "The present study has several limitations. The retrospective design relied on medical record documentation that demonstrated substantial missingness within symptom and complication variables. Detailed completeness assessment and proportions of missing data across study variables are summarized in S1 Table. Echocardiographic severity parameters, surgical history, socioeconomic conditions, and detailed dietary assessments were not consistently available for analysis. The study also originated from a single tertiary referral center, which may limit generalizability to broader community populations. Despite these limitations, the study included a relatively large outpatient cohort and integrated nutritional assessment, healthcare utilization analysis, and multivariable modeling within a pediatric congenital heart disease population from a resource limited setting."
?	Conflict of interest statement	ⓘ It is important to supply a conflict of interest statement, even if there are none to declare, to transparently present the research.
✓	Funding statement	ⓘ Thank you for reporting your funding source(s), or stating that there was no specific funding for this work.
n/a	Registration statement	ⓘ A registered protocol makes the planned research project more transparent and can protect against bias. Please consider registering future confirmatory studies.

Generated by ODDPub, limitation-recognizer, rtransparent, trial-identifier.

Figures

✓	No bar graphs of continuous data	ⓘ Thank you for not using bar graphs to present continuous data. Many different datasets and data distributions can lead to the same bar graph, and the actual data may suggest different conclusions from the summary statistics alone.
✓	No rainbow color maps	ⓘ Thank you for not using rainbow colormaps. Rainbow color maps distort readers' perception of

	the data by introducing visual artifacts and are not accessible to colorblind readers.

Generated by JetFighter, Barzooka.
