# Supplementary material for: Nutritional status, clinical burden, and healthcare utilization among pediatric outpatients with congenital heart disease: A retrospective cross-sectional study from Indonesia
Source: PLoS One. 2026 Oct 8;21(10):e0351014. doi: 10.1371/journal.pone.0351014 (PMC13648971; doi:10.1371/journal.pone.0351014)
Supplement: Supplementary file 8 [file pone.0351014.s007.docx]

Response to Editorial Request

We thank the editorial office for the clarification request. The Methods section (Ethical consideration subsection) has been revised to explicitly report the dates during which medical records were accessed for research purposes in DD/MM/YYYY format, in accordance with PLOS ONE submission requirements.

Specifically, we added the following sentence:

“Medical records were accessed for research purposes between 17/07/2025 and 31/12/2025.”
