# Supplementary material for: Nutritional status, clinical burden, and healthcare utilization among pediatric outpatients with congenital heart disease: A retrospective cross-sectional study from Indonesia
Source: PLoS One. 2026 Oct 8;21(10):e0351014. doi: 10.1371/journal.pone.0351014 (PMC13648971; doi:10.1371/journal.pone.0351014)
Supplement: Supplementary file 10 [file pone.0351014.s011.docx]

**Response to Reviewer #3**

We thank the reviewer for the careful assessment and constructive comments regarding the interpretation and clinical implications of our findings. We have revised the manuscript to improve the distinction between primary multivariable findings, univariable and sensitivity analyses, to temper causal interpretation, to acknowledge uncertainty related to missing clinical information and residual confounding, and to make the clinical implications more specific and directly linked to the observed findings.

**Comment 1**

**The conclusion states that malnutrition was associated with increased healthcare utilization, yet this was not demonstrated in the final multivariable model.**

**Response**

We agree that the previous wording did not sufficiently distinguish the primary multivariable analysis from the univariable and sensitivity analyses. In the primary multivariable model, healthcare utilization was not included as a covariate. Healthcare utilization was associated with malnutrition in the univariable analysis (crude OR 1.58, 95% CI 1.12–2.22; p = 0.009) and remained statistically significant in the sensitivity model that incorporated the original age-group classification (adjusted OR 1.54, 95% CI 1.07–2.22; p = 0.020). Therefore, we clarified the reporting so that healthcare utilization is not presented as an independent finding of the primary multivariable model.

We revised the Results section to explicitly identify healthcare utilization as a finding from the sensitivity model and to report its adjusted estimate. We also revised the Discussion and Conclusion to distinguish this finding from the primary multivariable results. No new statistical analysis was required because the sensitivity analysis had already included healthcare utilization and the corresponding estimates are reported in Supplementary Table S3.

**Changes made:**

We agree that the previous wording did not sufficiently distinguish the primary multivariable analysis from the univariable and sensitivity analyses. In the primary multivariable model, healthcare utilization was not included as a covariate. Healthcare utilization was associated with malnutrition in the univariable analysis (crude OR 1.58, 95% CI 1.12–2.22; p = 0.009) and remained statistically significant in the sensitivity model incorporating the original age-group classification (adjusted OR 1.54, 95% CI 1.07–2.22; p = 0.020). We therefore revised the manuscript to clarify that healthcare utilization was not an independent finding of the primary multivariable model. Specifically, the Results section now identifies healthcare utilization as a significant finding in the sensitivity model and reports its adjusted estimate, while the Discussion and Conclusion distinguish this finding from the primary multivariable results. No new statistical analysis was required because the sensitivity analysis had already included healthcare utilization and the corresponding estimates are reported in Supplementary Table S3.

**Comment 2**

**Some wording implies stronger inference than can be supported by a retrospective cross-sectional analysis.**

**Response**

We agree. We revised the manuscript to ensure that the findings are presented as observational associations rather than causal relationships or demonstrated intervention effects. In the Conclusion, the primary findings are now described using the more precise formulation that younger age groups and higher cumulative clinical burden were independently associated with greater odds of malnutrition in the primary multivariable model. We also added an explicit statement that the findings should be interpreted cautiously because of the retrospective cross-sectional design.

In addition, causal or outcome-promising language was removed from the clinical implications. Statements suggesting that nutritional surveillance or integrated outpatient management would improve growth outcomes or reduce disease burden were replaced with more cautious wording focused on identification of patients who may warrant closer nutritional assessment and multidisciplinary follow-up.

**Comment 3**

**The conclusion does not sufficiently acknowledge uncertainty caused by missing data and unmeasured confounders.**

**Response**

We agree and have added an explicit acknowledgment of these sources of uncertainty to the Conclusion. The revised Conclusion now states that the findings should be interpreted cautiously because of the retrospective cross-sectional design, substantial missingness in clinical variables, and potential residual confounding from unmeasured disease severity.

This wording is consistent with the limitations described in the Discussion. Symptom and complication variables had substantial missingness in the retrospective records, while detailed hemodynamic measures of disease severity were not consistently available and could not be incorporated into the primary multivariable model. The final primary regression covariates themselves had complete data; therefore, the revised wording does not imply that missingness occurred in the variables included in the primary regression model.

The corresponding completeness information is provided in Supplementary Table S1.

**Comment 4**

**Recommendations for practice are somewhat broad and not directly linked to specific interventions.**

**Response**

We agree with the reviewer and have revised the clinical recommendations throughout the manuscript to make them more specific and directly linked to the observed findings. Rather than broadly recommending integrated long-term outpatient management or implying that such care would improve growth outcomes or reduce disease burden, we now emphasize routine nutritional screening, particularly among younger children and those with greater cumulative clinical burden, followed by closer nutritional assessment and multidisciplinary follow-up where clinically indicated. This revised recommendation has been applied consistently across the Abstract, Discussion, and Conclusion, and the wording has been modified to avoid implying that intervention effectiveness was demonstrated by this retrospective cross-sectional study. In the Abstract, the previous statement that early nutritional surveillance and integrated long-term outpatient management may improve growth outcomes and reduce chronic disease burden was replaced with a recommendation for routine nutritional screening and closer nutritional assessment among younger children and those with greater clinical burden. In the Discussion, the broad recommendation for integrated long-term outpatient management and the statement regarding reducing complications and improving long-term growth outcomes were replaced with a more specific recommendation for nutritional screening and closer assessment in these higher-risk groups. The Conclusion was revised consistently to state that routine nutritional screening may help identify patients who warrant closer nutritional assessment and multidisciplinary follow-up. These revisions improve the specificity and clinical relevance of the recommendations while keeping the interpretation proportionate to the observational study design.
