# Supplementary material for: Nutritional status, clinical burden, and healthcare utilization among pediatric outpatients with congenital heart disease: A retrospective cross-sectional study from Indonesia
Source: PLoS One. 2026 Oct 8;21(10):e0351014. doi: 10.1371/journal.pone.0351014 (PMC13648971; doi:10.1371/journal.pone.0351014)
Supplement: Supplementary file 11 [file pone.0351014.s012.docx]

**RESPONSE TO ACADEMIC EDITOR**

**PLOS ONE | Manuscript PONE-D-26-24373R2**

| **Manuscript title** | Nutritional status, clinical burden, and healthcare utilization among pediatric outpatients with congenital heart disease: A retrospective cross-sectional study from Indonesia |
| --- | --- |
| **Authors** | Putri Amelia, Lourents Christian Dika Sahertian, Rizky Adriansyah, Johnson Kannady |
| **Journal** | PLOS ONE |

Dear Dr. Hany Mahmoud Abo-Haded and Editorial Team,

Thank you for the opportunity to further revise our manuscript and for the clear guidance provided in the latest editorial decision. We have carefully revised the Conclusion to address all four points raised in the Additional Editor Comments. We have also revisited the concerns raised by Reviewer #3 in the previous review round. Those concerns had already been addressed in the preceding revision, and we have now made the corresponding distinctions and limitations even more explicit in the Conclusion to ensure that the final interpretation remains fully aligned with the study design and statistical analyses.

**Response to the Additional Editor Comments**

| **Editor comment** | **Response and revision** |
| --- | --- |
| 1. Use more cautious language that reflects the limitations of the retrospective cross-sectional design. | We agree. We revised the Conclusion to avoid causal or temporal interpretations. The revised text states that the findings should be interpreted cautiously because the retrospective cross-sectional design does not establish temporal or causal relationships. Throughout the Conclusion, we use association language rather than causal wording. |
| 2. Provide more specific and actionable practice recommendations that are directly informed by the study findings. | We agree. We replaced the previous broad recommendation with a more specific practice implication. The revised Conclusion recommends routine nutritional screening during pediatric CHD follow-up, with particular attention to younger children and those with greater documented clinical burden. We further state that this approach may facilitate earlier nutritional assessment and appropriate multidisciplinary management. These recommendations directly reflect the factors associated with malnutrition in the primary multivariable model. |
| 3. Explicitly acknowledge the potential impact of missing data and unmeasured confounding on the results. | We agree. The revised Conclusion now explicitly states that missing clinical data and unmeasured disease severity may have influenced the observed associations. This wording acknowledges the uncertainty introduced by substantial missingness in clinical variables and by important disease severity factors that were not available for adjustment in the retrospective dataset without overstating the direction or magnitude of potential bias. |
| 4. Clearly distinguish between findings observed in unadjusted analyses and those retained in the final multivariable model. | We agree and have made this distinction explicit. The revised Conclusion identifies younger age groups and higher cumulative clinical burden as the factors associated with greater odds of malnutrition in the primary multivariable model. It separately states that healthcare utilization was associated with malnutrition in univariable analysis and remained statistically significant in the sensitivity analysis, but was not retained in the primary multivariable model. This revision prevents the healthcare utilization finding from being interpreted as an independent association in the primary model. |

Relationship to the Previous Reviewer Comments

We also note that the four latest editorial points closely correspond to the Conclusion concerns raised by Reviewer #3 in the previous review round. Specifically, Reviewer #3 previously requested more cautious wording for the retrospective cross-sectional design, clearer acknowledgment of uncertainty from missing data and unmeasured confounders, more actionable practice recommendations, and correction of the interpretation of healthcare utilization because that association was not demonstrated in the final multivariable model. Reviewer #3 also described the Conclusion as somewhat long and repetitive. We addressed these concerns in the previous revision and have now further refined the Conclusion so that each issue is stated explicitly and concisely. The present revision therefore does not change the underlying findings. Instead, it strengthens the transparency and precision of their interpretation.

Revised Conclusion

*Pediatric outpatients with congenital heart disease at Adam Malik General Hospital had a substantial burden of malnutrition, with nearly half of the cohort classified as underweight or severely underweight. Younger age groups and higher cumulative clinical burden were associated with greater odds of malnutrition in the primary multivariable model. Healthcare utilization was associated with malnutrition in univariable analysis and remained statistically significant in the sensitivity analysis, but it was not retained in the primary multivariable model. These findings should be interpreted cautiously because the retrospective cross-sectional design does not establish temporal or causal relationships, while missing clinical data and unmeasured disease severity may have influenced the observed associations. In practice, routine nutritional screening during pediatric CHD follow-up, with particular attention to younger children and those with greater documented clinical burden, may facilitate earlier nutritional assessment and appropriate multidisciplinary management. Prospective studies using standardized nutritional and clinical severity measures are needed to confirm these associations.*

Location of Revision

The revised Conclusion has been incorporated into the revised manuscript. The corresponding changes are highlighted in the marked-up manuscript. We have retained the primary findings and revised the wording to improve statistical precision, limitation-aware interpretation, and the specificity of the clinical recommendation.

Closing

We appreciate the Academic Editor’s guidance, which helped us further improve the clarity and scientific precision of the manuscript. We believe that the revised Conclusion now accurately reflects the observational study design, clearly differentiates univariable and multivariable findings, acknowledges the principal sources of uncertainty, and provides practice recommendations that are directly informed by the study findings.

Sincerely,

Putri Amelia, MD

on behalf of all authors
