# Supplementary material for: Nutritional status, clinical burden, and healthcare utilization among pediatric outpatients with congenital heart disease: A retrospective cross-sectional study from Indonesia
Source: PLoS One. 2026 Oct 8;21(10):e0351014. doi: 10.1371/journal.pone.0351014 (PMC13648971; doi:10.1371/journal.pone.0351014)
Supplement: Peer review history

# Author comment: Author response to Decision Letter 0

**DOI:** 10.1371/journal.pone.0351014.r001

27 May 2026

Attachment Submitted filename: RESPONSE TO EDITORIAL.docx

# Peer review: Decision Letter 0

**DOI:** 10.1371/journal.pone.0351014.r002

**Authors:** Hany Mahmoud Abo-Haded

14 Jul 2026

Dear Dr. Amelia,

Thank you for submitting your manuscript to PLOS ONE. After careful consideration, we feel that it has merit but does not fully meet PLOS ONE’s publication criteria as it currently stands. Therefore, we invite you to submit a revised version of the manuscript that addresses the points raised during the review process.

Please submit your revised manuscript by Sep 12 2026 11:59PM. If you will need more time than this to complete your revisions, please reply to this message or contact the journal office at plosone@plos.org. When you're ready to submit your revision, log on to https://www.editorialmanager.com/pone/ and select the 'Submissions Needing Revision' folder to locate your manuscript file.

- A letter that responds to each point raised by the academic editor and reviewer(s). You should upload this letter as a separate file labeled 'Response to Reviewers'.
- A marked-up copy of your manuscript that highlights changes made to the original version. You should upload this as a separate file labeled 'Revised Manuscript with Track Changes'.
- An unmarked version of your revised paper without tracked changes. You should upload this as a separate file labeled 'Manuscript'.

If you would like to make changes to your financial disclosure, please include your updated statement in your cover letter. Guidelines for resubmitting your figure files are available below the reviewer comments at the end of this letter.

If applicable, we recommend that you deposit your laboratory protocols in protocols.io to enhance the reproducibility of your results. Protocols.io assigns your protocol its own identifier (DOI) so that it can be cited independently in the future. For instructions see: https://journals.plos.org/plosone/s/submission-guidelines#loc-laboratory-protocols. Additionally, PLOS ONE offers an option for publishing peer-reviewed Lab Protocol articles, which describe protocols hosted on protocols.io. Read more information on sharing protocols at https://plos.org/protocols?utm_medium=editorial-email&utm_source=authorletters&utm_campaign=protocols.

As the corresponding author, your ORCID iD is verified in the submission system and will appear in the published article. PLOS supports the use of ORCID, and we encourage all coauthors to register for an ORCID iD and use it as well. Please encourage your coauthors to verify their ORCID iD within the submission system before final acceptance, as unverified ORCID iDs will not appear in the published article. *Only*  the individual author can complete the verification step; PLOS staff *cannot*  verify ORCID iDs on behalf of authors.

We look forward to receiving your revised manuscript.

Kind regards,

Hany Mahmoud Abo-Haded, MD

Academic Editor

PLOS One

Journal Requirements:

When submitting your revision, we need you to address these additional requirements.

1. Please ensure that your manuscript meets PLOS ONE's style requirements, including those for file naming. The PLOS ONE style templates can be found at

https://journals.plos.org/plosone/s/file?id=wjVg/PLOSOne_formatting_sample_main_body.pdf and https://journals.plos.org/plosone/s/file?id=ba62/PLOSOne_formatting_sample_title_authors_affiliations.pdf

2. Your ethics statement should only appear in the Methods section of your manuscript. If your ethics statement is written in any section besides the Methods, please delete it from any other section.

3. In the online submission form, you indicated that the data underlying the findings of this study contain potentially identifiable patient information derived from retrospective medical records and cannot be shared publicly because of institutional and ethical restrictions. Deidentified data may be made available from the corresponding author upon reasonable request and with permission from Adam Malik General Hospital and the Health Research Ethics Committee of the Faculty of Medicine, Universitas Sumatera Utara.

All PLOS journals now require all data underlying the findings described in their manuscript to be freely available to other researchers, either 1. In a public repository, 2. Within the manuscript itself, or 3. Uploaded as supplementary information.

This policy applies to all data except where public deposition would breach compliance with the protocol approved by your research ethics board. If your data cannot be made publicly available for ethical or legal reasons (e.g., public availability would compromise patient privacy), please explain your reasons on resubmission and your exemption request will be escalated for approval.

4. Please upload a copy of Supporting Information Figure S1 which you refer to in your text on page 14, 20.

5. If the reviewer comments include a recommendation to cite specific previously published works, please review and evaluate these publications to determine whether they are relevant and should be cited. There is no requirement to cite these works unless the editor has indicated otherwise.

[Note: HTML markup is below. Please do not edit.]

Reviewers' comments:

Reviewer's Responses to Questions

**Comments to the Author**

1. Is the manuscript technically sound, and do the data support the conclusions?

Reviewer #1: Yes

Reviewer #2: Yes

Reviewer #3: Partly

Reviewer #4: Yes

**********

2. Has the statistical analysis been performed appropriately and rigorously?

Reviewer #1: Yes

Reviewer #2: Yes

Reviewer #3: Yes

Reviewer #4: Yes

**********

3. Have the authors made all data underlying the findings in their manuscript fully available?

The PLOS Data policy

Reviewer #1: Yes

Reviewer #2: Yes

Reviewer #3: Yes

Reviewer #4: No

**********

4. Is the manuscript presented in an intelligible fashion and written in standard English?

Reviewer #1: Yes

Reviewer #2: Yes

Reviewer #3: Yes

Reviewer #4: Yes

**********

Reviewer #1: This is an automated report for PONE-D-26-24373. This report was solicited by the PLOS One editorial team and provided by ScreenIT. ScreenIT is an independent group of scientists developing automated tools that analyze academic papers. A set of automated tools screened your submitted manuscript and provided the report below. Each tool was created by your academic colleagues with the goal of helping authors. The tools look for factors that are important for transparency, rigor and reproducibility, and we hope that the report might help you to improve reporting in your manuscript. Within the report you will find links to more information about the items that the tools check. These links include helpful papers, websites, or videos that explain why the item is important. While our screening tools aim to improve and maintain quality standards they may, on occasion, miss nuances specific to your study type or flag something incorrectly. Each tool has limitations that are described on the ScreenIT website. The tools screen the main file for the paper; they are not able to screen supplements stored in separate files. Please note that the Academic Editor had access to these comments while making a decision on your manuscript. The Academic Editor may ask that issues flagged in this report be addressed. If you would like to provide feedback on the ScreenIT tool, please email the team at ScreenIt@bih-charite.de. If you have questions or concerns about the review process, please contact the PLOS One office at plosone@plos.org.

Reviewer #2: The manuscript addresses an important clinical issue and provides valuable data from Indonesia. Addressing these methodological and reporting issues would improve transparency, reproducibility, and the overall scientific contribution of the study.

Reviewer #3: Thank you for your efforts

1-. Introduction : Provides clear clinical background on CHD and its global burden. But ; some parts are slightly verbose and could be more concise and uimited mention of specific prior quantitative data to benchmark the problem.

2-Methods : There is well-defined inclusion/exclusion criteria and appropriate operational definitions (malnutrition, CHD classification). However , as retrospective design limits causal inference. High missingness in clinical variables, with no imputation. Limited variables (no disease severity, socioeconomic or dietary data) as well as the burden score, although useful, is not externally validated.

3- Results : Well-structured presentation (tables, figures, logical flow) are provided with clear reporting of baseline characteristics and CHD distribution. However: missing clinical data: High missingness in symptoms/complications may introduce bias (complete-case analysis). Limited variables: Lack of key factors such as: disease severity (echocardiographic details),surgical/intervention history and socioeconomic and dietary information. Also still weakness regarding the some findings are descriptive without deeper statistical comparison (e.g., between CHD subtypes). Small subgroup sizes (e.g., cyanotic CHD) reduce robustness.The burden score distribution is skewed, limiting interpretability

4-Discussion : Interprets findings in line with existing literature and provides physiological explanations for malnutrition.Need to avoid repetition of some explanations of results. There is limited discussion of unexpected findings (e.g., non-significant cyanotic CHD). Could better explore health system implications or policy relevance

5-Conclusion : Slightly long and repetitive. Could include a more actionable final recommendation.

Reviewer #4: The manuscript addresses an important topic which is the burden of malnutrition among children with CHD in a low-resource country and includes a relatively large outpatient cohort (n = 606). The study is clinically relevant and provides useful epidemiological data from Indonesia, where such information is limited. However, there are several important methodological and reporting issues that should be addressed before the manuscript can be considered for publication.

1-Nutritional assessment is inadequately defined. This is the most important weakness. The authors state that nutritional status was assessed using: “WHO growth standards and age-appropriate weight-for-age evaluation.” However, they never specify which anthropometric indices were used; WHO 2006 or WHO 2007 references, whether z-scores were calculated

the exact cutoffs for underweight/severe underweight, whether BMI-for-age or weight-for-height was used in older children. Weight-for-age is not recommended for children older than approximately 10 years and is inappropriate for adolescents.

The authors should clearly describe: anthropometric index, reference standards, z-score thresholds

age-specific assessment methods.

2. “Burden score” lacks validation. The cumulative burden score is one of the main predictors in the regression.however:

it is entirely investigator-created, no rationale is provided, no validation, andno reproducibility assessment.

The authors should justify construction of this score or acknowledge its exploratory nature more explicitly.

3. Confounding by disease severity.The regression model adjusts only for age, sex, CHD physiology, and burden score.

It does not account for important confounders such as surgical status, repaired vs unrepaired lesions, defect size, heart failure severity, pulmonary hypertension severity, socioeconomic status, genetic syndromes, prematurity.

4. Cross-sectional design limits causal interpretation.Several statements imply causality such as "healthcare utilization increased because of malnutrition" or "malnutrition leads to greater clinical burden"

Because exposure and outcome were measured simultaneously, only associations—not causal relationships—can be inferred.

This wording should be revised throughout the manuscript.

5. Missing data: The authors acknowledge substantial missingness in symptoms and complications but do not report: percentage missing for each variable, which variables entered regression, number of patients excluded from complete-case analysis.

A flow diagram showing exclusions from regression would improve transparency.

6.Small cyanotic subgroup: Only 31 patients had cyanotic CHD (5.1%). Consequently, statistical power is limited, adjusted OR estimates are unstable, the nonsignificant findings should not be interpreted as evidence of no association.

This limitation should be emphasized.

7- Classification of CHD: Grouping lesions simply as cyanotic and acyanotic oversimplifies disease severity. for example,

large VSD and small VSD are biologically very different. Hemodynamic significance would have been more informative.

8- Age categories: Combining adolescents with neonates as the regression reference group is statistically convenient but clinically illogical. These populations are fundamentally different. Alternative modeling approaches should be considered.

9- Discussion: The Discussion largely repeats textbook mechanisms. It would benefit from more comparison with previous Southeast Asian studies, more emphasis on why Indonesian findings differ, discussion of tertiary referral bias.

10-Generalizability: This is a single-center tertiary referral hospital.The study population likely includes more complex CHD than community hospitals. Generalizability should be discussed more explicitly.

11- Introduction: The Introduction is somewhat long and contains several paragraphs describing basic CHD physiology that most readers already know. It could be shortened.

12- Methods: Specify ICD coding method, echocardiographic diagnostic criteria, who performed nutritional assessment.

13- Tables:

Table 1 duplicates nutritional status and malnutrition status. One of these could be removed.

14- Figures: Figure legends are generally good. However, Figure 2 would benefit from including exact percentages.

**********

what does this mean?). If published, this will include your full peer review and any attached files.

If you choose “no”, your identity will remain anonymous but your review may still be made public.

**Do you want your identity to be public for this peer review?**  For information about this choice, including consent withdrawal, please see our Privacy Policy

Reviewer #1: No

Reviewer #2: No

Reviewer #3: **Yes:**  Safaa ELMeneza

Reviewer #4: **Yes:**  Doaa Mohamed El Amrousy

**********

[NOTE: If reviewer comments were submitted as an attachment file, they will be attached to this email and accessible via the submission site. Please log into your account, locate the manuscript record, and check for the action link "View Attachments". If this link does not appear, there are no attachment files.]

To ensure your figures meet our technical requirements, please review our figure guidelines: https://journals.plos.org/plosone/s/figures

You may also use PLOS’s free figure tool, NAAS, to help you prepare publication quality figures: https://journals.plos.org/plosone/s/figures#loc-tools-for-figure-preparation.

NAAS will assess whether your figures meet our technical requirements by comparing each figure against our figure specifications.

Attachment Submitted filename: report_10.64898+2026.05.23.26353925.pdf

Attachment Submitted filename: Comments.docx

# Author comment: Author response to Decision Letter 1

**DOI:** 10.1371/journal.pone.0351014.r003

7 Aug 2026

Response to Reviewers

Manuscript title: Nutritional status, clinical burden, and healthcare utilization among pediatric outpatients with congenital heart disease: A retrospective cross-sectional study from Indonesia

Journal: PLOS ONE

Dear Academic Editor and Reviewers,

We sincerely thank the Academic Editor and all reviewers for their careful evaluation of our manuscript and for the constructive suggestions. We have revised the manuscript, supplementary materials, and supporting tables to improve clarity, reporting completeness, and transparency. Below, we respond to each comment point-by-point. Reviewer comments are reproduced in abbreviated form, followed by our responses and the corresponding changes made in the revised submission.

Response to the Academic Editor and Journal Requirements

Comment: Please ensure that the manuscript meets PLOS ONE style requirements, including file naming.

Response: We have checked the revised submission package and aligned the manuscript and supporting files with the requested PLOS ONE formatting requirements. File names and supplementary materials have been organized for resubmission in the required format.

Changes made: The manuscript, revised manuscript with tracked changes, response letter, and supplementary files have been prepared as separate submission items.

Comment: Your ethics statement should only appear in the Methods section of your manuscript.

Response: We have retained the ethics statement only in the Methods section and removed any duplicate ethics wording from other parts of the manuscript.

Changes made: The revised Methods now contain the sole ethics statement for the study.

Comment: Please clarify the availability of the underlying data.

Response: We have updated the Data Availability Statement to indicate that the study dataset has been de-identified and that the underlying data are provided in a form that protects patient confidentiality. We also clarified that the data were derived from retrospective medical records and that any direct identifiers were removed before analysis.

Changes made: The revised statement reflects the institutional and ethical constraints while preserving transparency about data access.

Comment: Please upload a copy of Supporting Information Figure S1 which you refer to in your text.

Response: We have uploaded Supporting Information Figure S1 and ensured that it is referenced consistently in the Results and supplementary material.

Changes made: The figure accompanies the revised manuscript package.

Comment: Please review any cited works recommended by reviewers and include only those that are relevant.

Response: We reviewed the suggested references and retained only those that were relevant to the revised discussion and reporting. We did not add references that were not necessary for the manuscript’s scientific argument.

Changes made: The reference list was checked for relevance and consistency with the revised text.

Reviewer #1

Comment: Automated screening report from ScreenIT.

Response: We appreciate the automated screening report. We reviewed the reporting checklist carefully and revised the manuscript and supplementary files to improve completeness, transparency, and reproducibility where applicable.

Changes made: Key reporting items addressed in the revision include the methods description, missing-data reporting, data availability statement, and supplementary figure inclusion.

Reviewer #2

Comment: The manuscript addresses an important clinical issue and provides valuable data from Indonesia. Addressing methodological and reporting issues would improve transparency, reproducibility, and the overall scientific contribution of the study.

Response: We thank the reviewer for this positive assessment. In response, we revised the Methods, Results, Discussion, and supplementary materials to improve methodological clarity, reporting transparency, and reproducibility. We also strengthened the data availability language and clarified the handling of missing data and exploratory variables.

Changes made: The revised manuscript now presents the study design, variables, and supplementary completeness assessment more explicitly.

Reviewer #3

Comment: The Introduction is clear but could be more concise and include more targeted quantitative context.

Response: Thank you for this helpful suggestion. We revised the Introduction by removing repetitive background information and streamlining the clinical rationale to improve readability. We also strengthened the epidemiologic context by incorporating concise quantitative evidence from recent large-scale studies on the global burden of congenital heart disease and growth impairment, while refining the description of the Indonesian knowledge gap and the rationale for evaluating nutritional status, clinical burden, and healthcare utilization in a tertiary referral outpatient population.

Changes made: The Introduction was revised to (1) reduce redundant background information, (2) incorporate quantitative epidemiologic evidence to better contextualize the burden of congenital heart disease, (3) clarify the existing evidence gap in Indonesia, and (4) more clearly align the study rationale and objectives with the clinical importance of nutritional status and healthcare utilization in pediatric congenital heart disease.

Comment: The Methods are well-defined, but the retrospective design limits causal inference; there is high missingness in clinical variables, no imputation, and limited variables.

Response: Thank you for this important comment. We clarified that the retrospective study design supports assessment of associations rather than causal inference. We also expanded the description of missing data handling by specifying that missingness was primarily confined to symptom and complication variables, no imputation was performed because of the non-random pattern of missingness, and complete-case analysis was used for regression. In addition, we clarified that disease severity, socioeconomic characteristics, and dietary information were not consistently available in the medical records and therefore could not be included in the analyses.

Changes made: The Methods were revised to strengthen the descriptions of missing data handling and variable availability, S1 Table summarizes data completeness across study variables, and the Limitations section explicitly acknowledges the absence of disease severity, socioeconomic, and dietary variables.

Comment: The Results are well structured, but missingness, limited variables, the lack of deeper comparisons, and small subgroups reduce robustness.

Response: We thank the reviewer for this comment. We clarified the extent of missing data, reported data completeness, and acknowledged the limited statistical power of small subgroups. We also emphasized that subgroup findings are descriptive and that the analyses should be interpreted as exploratory.

Changes made: Relevant revisions were incorporated into the Results, with additional information provided in the supplementary tables (S1–S5), including data completeness, sensitivity analyses, and model diagnostics.

Comment: The Discussion repeats result explanations and could better address unexpected findings and health-system implications.

Response: Thank you for this valuable suggestion. We revised the Discussion to reduce repetition of descriptive result summaries and place greater emphasis on interpretation. Specifically, we expanded the discussion of the unexpected finding that cyanotic congenital heart disease was not independently associated with malnutrition after multivariable adjustment by comparing our findings with evidence from Southeast Asian studies and discussing potential explanations, including differences in study populations, outpatient versus surgical cohorts, and cumulative hemodynamic burden. We also incorporated a discussion of tertiary referral bias and explained how the role of Adam Malik General Hospital as a regional referral center may have influenced the observed prevalence of malnutrition, pulmonary hypertension, and healthcare utilization. Finally, we strengthened the discussion of the clinical and health-system implications by emphasizing the importance of early nutritional surveillance, multidisciplinary outpatient follow-up, and resource allocation for children with congenital heart disease in resource-limited settings.

Changes made: The Discussion has been revised to (1) reduce repetition of mechanistic explanations, (2) include comparisons with Indonesian and Southeast Asian studies, (3) provide a more detailed interpretation of the non-significant association between cyanotic CHD and malnutrition, (4) discuss the impact of tertiary referral bias on the study findings, and (5) expand the clinical and health-system implications for outpatient management in resource-limited settings.

Comment: The Conclusion is slightly long and repetitive and could end with a more actionable recommendation.

Response: We thank the reviewer for this suggestion. We shortened the Conclusion to emphasize the principal findings and strengthened the final statement with a more actionable recommendation on nutritional surveillance and multidisciplinary outpatient care.

Changes made: The Conclusion has been revised to improve conciseness and highlight the main clinical take-home message

Reviewer #4

Comment: Nutritional assessment is inadequately defined.

Response: We thank the reviewer for this comment. We revised the Methods to clearly specify the age-specific anthropometric assessment, reference standards, nutritional cutoffs, and outcome classification.

Changes made: The relevant methodological details have been added to the Methods section.

Comment: The burden score lacks validation.

Response: We agree that the burden score is investigator-developed and exploratory rather than externally validated. We revised the manuscript to state this explicitly and to caution readers that the score should be interpreted as a pragmatic summary of cumulative symptoms and complications, not as a validated clinical instrument.

Changes made: The Methods and Limitations now describe the burden score as exploratory and unvalidated.

Comment: Confounding by disease severity was not fully addressed.

Response: We thank the reviewer for this important comment. We clarified that detailed disease-severity variables were unavailable in the retrospective dataset and that residual confounding may remain.

Changes made: The Limitations now explicitly acknowledge the absence of detailed echocardiographic severity data and the potential for residual confounding.

Comment: Because this is a cross-sectional study, causal wording should be revised.

Response: We revised causal phrasing throughout the manuscript and now describe the findings as associations rather than causal effects. Statements implying that malnutrition causes higher utilization, or that utilization increased because of malnutrition, were rewritten to avoid causal inference.

Changes made: The Discussion and Conclusion now use association-based language consistently.

Comment: Missing data were not sufficiently transparent.

Response: We thank the reviewer for this comment. We clarified the completeness of study variables and the approach used to handle missing data

Changes made: S1 Table now summarizes missingness across variables, and the Methods describe the complete case analysis and the absence of data imputation.

Comment: The cyanotic subgroup is small and estimates are unstable.

Response: We agree. We now emphasize that the cyanotic subgroup is small and that the corresponding estimates should be interpreted cautiously. We avoid overinterpreting the non-significant findings as evidence of no association.

Changes made: The Results and Discussion now explicitly note the limited precision in the cyanotic subgroup.

Comment: Classification of CHD by cyanotic versus acyanotic physiology oversimplifies disease severity.

Response: We acknowledge this limitation. CHD was classified as cyanotic or non-cyanotic, the most reproducible classification available because lesion-level hemodynamic severity data were not consistently available in the retrospective records. We have clarified this in the manuscript.

Changes made: The rationale for using cyanotic versus non-cyanotic classification is now clarified in the Methods and acknowledged as a limitation in the Discussion.

Comment: Age categories combine clinically different groups, and the reference group is not ideal.

Response: We agree. Because of sparse observations in the extreme age groups, neonatal and adolescent categories were combined in the primary model to improve model stability. We also performed a sensitivity analysis using the original age-group classification, which yielded consistent results.

Changes made: The Methods now clarify the rationale for the grouped age categories and the sensitivity analysis has been added to the Supplementary Material.

Comment: The Discussion is repetitive and should compare findings more strongly with Southeast Asian literature and referral bias.

Response: We revised the Discussion to reduce repetition and expanded comparison with regional and international literature. We also added a clearer explanation of why findings from a tertiary referral hospital may reflect referral bias and may not be directly generalizable to community settings.

Changes made: The revised Discussion now includes a more explicit interpretation of regional context and tertiary-care bias.

Comment: Generalizability should be discussed more explicitly.

Response: We added a stronger statement that the study reflects a single tertiary referral center in Indonesia and therefore may overrepresent more complex cases. We note that the findings are most directly applicable to similar referral-based outpatient populations.

Changes made: The Limitations now explicitly address external validity.

Comment: The Introduction is too long and still contains extensive general background on congenital heart disease. It should be shortened further while maintaining the study rationale.

Response: We agree and have further streamlined the Introduction by removing redundant background information while retaining the essential scientific rationale, study context, and objective.

Changes made: The Introduction has been shortened from three paragraphs to two more concise paragraphs, with reduced general background on CHD and greater emphasis on the knowledge gap, study rationale, and study objective.

Comment: Methods should specify ICD coding, echocardiographic criteria, and who performed nutritional assessment.

Response: We revised the Methods to clarify patient identification using hospital diagnostic records, echocardiographic confirmation of CHD, and the procedures used for routine nutritional assessment.

Changes made: Additional details on patient identification, echocardiographic confirmation, and nutritional assessment have been added to the Methods section.

Comment: Table 1 duplicates nutritional status and malnutrition status.

Response: We reviewed the tables and retained a single clear presentation of nutritional categories so that the table layout is less repetitive and easier to interpret.

Changes made: The table presentation has been streamlined accordingly.

Comment: Figure 2 would benefit from including exact percentages.

Response: We revised the Figure 2 legend to include the exact percentages for each nutritional status category across age groups.

Changes made: Figure 2 and its legend were updated for clarity.

We again thank the Academic Editor and the reviewers for their helpful comments. We believe the revisions have substantially improved the manuscript’s clarity, transparency, and interpretability. We hope the revised version is now suitable for further consideration.

Sincerely,

The Authors

Attachment Submitted filename: RESPONSE_TO_EDITORIAL_auresp_1.docx

# Peer review: Decision Letter 1

**DOI:** 10.1371/journal.pone.0351014.r004

**Authors:** Hany Mahmoud Abo-Haded

31 Aug 2026

Dear Dr. Amelia,

Thank you for submitting your manuscript to PLOS One. After careful consideration, we feel that it has merit but does not fully meet PLOS One’s publication criteria as it currently stands. Therefore, we invite you to submit a revised version of the manuscript that addresses the points raised during the review process.

Please submit your revised manuscript by Sep 30 2026 11:59PM. If you will need more time than this to complete your revisions, please reply to this message or contact the journal office at plosone@plos.org. When you're ready to submit your revision, log on to https://www.editorialmanager.com/pone/ and select the 'Submissions Needing Revision' folder to locate your manuscript file.

- A letter that responds to each point raised by the academic editor and reviewer(s). You should upload this letter as a separate file labeled 'Response to Reviewers'.
- A marked-up copy of your manuscript that highlights changes made to the original version. You should upload this as a separate file labeled 'Revised Manuscript with Track Changes'.
- An unmarked version of your revised paper without tracked changes. You should upload this as a separate file labeled 'Manuscript'.

If you would like to make changes to your financial disclosure, please include your updated statement in your cover letter. Guidelines for resubmitting your figure files are available below the reviewer comments at the end of this letter.

If applicable, we recommend that you deposit your laboratory protocols in protocols.io to enhance the reproducibility of your results. Protocols.io assigns your protocol its own identifier (DOI) so that it can be cited independently in the future. For instructions see: https://journals.plos.org/plosone/s/submission-guidelines#loc-laboratory-protocols. Additionally, PLOS One offers an option for publishing peer-reviewed Lab Protocol articles, which describe protocols hosted on protocols.io. Read more information on sharing protocols at https://plos.org/protocols?utm_medium=editorial-email&utm_source=authorletters&utm_campaign=protocols.

As the corresponding author, your ORCID iD is verified in the submission system and will appear in the published article. PLOS supports the use of ORCID, and we encourage all coauthors to register for an ORCID iD and use it as well. Please encourage your coauthors to verify their ORCID iD within the submission system before final acceptance, as unverified ORCID iDs will not appear in the published article. *Only*  the individual author can complete the verification step; PLOS staff *cannot*  verify ORCID iDs on behalf of authors.

We look forward to receiving your revised manuscript.

Kind regards,

Hany Mahmoud Abo-Haded, MD

Academic Editor

PLOS One

Journal Requirements:

If the reviewer comments include a recommendation to cite specific previously published works, please review and evaluate these publications to determine whether they are relevant and should be cited. There is no requirement to cite these works unless the editor has indicated otherwise.

Please review your reference list to ensure that it is complete and correct. If you have cited papers that have been retracted, please include the rationale for doing so in the manuscript text, or remove these references and replace them with relevant current references. Any changes to the reference list should be mentioned in the rebuttal letter that accompanies your revised manuscript. If you need to cite a retracted article, indicate the article’s retracted status in the References list and also include a citation and full reference for the retraction notice.

[Note: HTML markup is below. Please do not edit.]

Reviewers' comments:

Reviewer's Responses to Questions

**Comments to the Author**

Reviewer #3: All comments have been addressed

Reviewer #4: All comments have been addressed

**********

2. Is the manuscript technically sound, and do the data support the conclusions?

Reviewer #3: Partly

Reviewer #4: Yes

**********

3. Has the statistical analysis been performed appropriately and rigorously?

Reviewer #3: Yes

Reviewer #4: Yes

**********

4. Have the authors made all data underlying the findings in their manuscript fully available?

The PLOS Data policy

Reviewer #3: Yes

Reviewer #4: No

**********

5. Is the manuscript presented in an intelligible fashion and written in standard English?

Reviewer #3: Yes

Reviewer #4: Yes

**********

Reviewer #3: The conclusion needs improvement:

- States that malnutrition was associated with increased healthcare utilization, yet this was not demonstrated in the final multivariable model.

- Some wording implies stronger inference than can be supported by a retrospective cross-sectional analysis.

- The conclusion does not sufficiently acknowledge uncertainty caused by missing data and unmeasured confounders.

-Recommendations for practice are somewhat broad and not directly linked to specific interventions.

To improve, I suggest :

- States that malnutrition was associated with increased healthcare utilization, yet this was not demonstrated in the final multivariable model.

-Some wording implies stronger inference than can be supported by a retrospective cross-sectional analysis.

-The conclusion does not sufficiently acknowledge uncertainty caused by missing data and unmeasured confounders.

-Recommendations for practice are somewhat broad and not directly linked to specific interventions.

Reviewer #4: The authors performed the required changes. The manuscript greatly improved and is now ready for publication.

**********

what does this mean?). If published, this will include your full peer review and any attached files.

If you choose “no”, your identity will remain anonymous but your review may still be made public.

**Do you want your identity to be public for this peer review?**  For information about this choice, including consent withdrawal, please see our Privacy Policy

Reviewer #3: **Yes:**  Safaa ELMeneza

Reviewer #4: No

**********

[NOTE: If reviewer comments were submitted as an attachment file, they will be attached to this email and accessible via the submission site. Please log into your account, locate the manuscript record, and check for the action link "View Attachments". If this link does not appear, there are no attachment files.]

To ensure your figures meet our technical requirements, please review our figure guidelines: https://journals.plos.org/plosone/s/figures

You may also use PLOS’s free figure tool, NAAS, to help you prepare publication quality figures: https://journals.plos.org/plosone/s/figures#loc-tools-for-figure-preparation.

NAAS will assess whether your figures meet our technical requirements by comparing each figure against our figure specifications.

# Author comment: Author response to Decision Letter 2

**DOI:** 10.1371/journal.pone.0351014.r005

2 Sep 2026

We sincerely thank the reviewer for the constructive comments. We have revised the manuscript to address all points raised. We clarified that healthcare utilization was associated with malnutrition in the univariable analysis and remained significant in the sensitivity analysis, but was not included in the primary multivariable model. We revised the manuscript to present the findings as observational associations and removed wording that could imply causal relationships or demonstrated intervention effects. We also added explicit acknowledgment of substantial missingness in clinical variables, unavailable measures of disease severity, and potential residual confounding. Finally, we revised the clinical recommendations to focus on routine nutritional screening and closer nutritional assessment and multidisciplinary follow-up for younger children and those with greater clinical burden, without implying demonstrated treatment effects. We believe these revisions have addressed all of the reviewer’s concerns and improved the clarity and appropriate interpretation of the manuscript.

Attachment Submitted filename: Response to reviewer.docx

# Editor report: Decision Letter 2

**DOI:** 10.1371/journal.pone.0351014.r006

**Authors:** Hany Mahmoud Abo-Haded

17 Sep 2026

Nutritional status, clinical burden, and healthcare utilization among pediatric outpatients with congenital heart disease: A retrospective cross-sectional study from Indonesia

PLOS One

Dear Dr. Amelia,

Thank you for submitting your manuscript to PLOS One. After careful consideration, we feel that it has merit but does not fully meet PLOS One’s publication criteria as it currently stands. Therefore, we invite you to submit a revised version of the manuscript that addresses the points raised during the review process.

Please submit your revised manuscript by Oct 17 2026 11:59PM. If you will need more time than this to complete your revisions, please reply to this message or contact the journal office at plosone@plos.org. When you're ready to submit your revision, log on to https://www.editorialmanager.com/pone/ and select the 'Submissions Needing Revision' folder to locate your manuscript file.

- A letter that responds to each point raised by the academic editor and reviewer(s). You should upload this letter as a separate file labeled 'Response to Reviewers'.
- A marked-up copy of your manuscript that highlights changes made to the original version. You should upload this as a separate file labeled 'Revised Manuscript with Track Changes'.
- An unmarked version of your revised paper without tracked changes. You should upload this as a separate file labeled 'Manuscript'.

If you would like to make changes to your financial disclosure, please include your updated statement in your cover letter. Guidelines for resubmitting your figure files are available below the reviewer comments at the end of this letter.

If applicable, we recommend that you deposit your laboratory protocols in protocols.io to enhance the reproducibility of your results. Protocols.io assigns your protocol its own identifier (DOI) so that it can be cited independently in the future. For instructions see: https://journals.plos.org/plosone/s/submission-guidelines#loc-laboratory-protocols. Additionally, PLOS One offers an option for publishing peer-reviewed Lab Protocol articles, which describe protocols hosted on protocols.io. Read more information on sharing protocols at https://plos.org/protocols?utm_medium=editorial-email&utm_source=authorletters&utm_campaign=protocols.

As the corresponding author, your ORCID iD is verified in the submission system and will appear in the published article. PLOS supports the use of ORCID, and we encourage all coauthors to register for an ORCID iD and use it as well. Please encourage your coauthors to verify their ORCID iD within the submission system before final acceptance, as unverified ORCID iDs will not appear in the published article. *Only*  the individual author can complete the verification step; PLOS staff *cannot*  verify ORCID iDs on behalf of authors.

We look forward to receiving your revised manuscript.

Kind regards,

Hany Mahmoud Abo-Haded, MD

Academic Editor

PLOS One

Journal Requirements:

If the reviewer comments include a recommendation to cite specific previously published works, please review and evaluate these publications to determine whether they are relevant and should be cited. There is no requirement to cite these works unless the editor has indicated otherwise.

Please review your reference list to ensure that it is complete and correct. If you have cited papers that have been retracted, please include the rationale for doing so in the manuscript text, or remove these references and replace them with relevant current references. Any changes to the reference list should be mentioned in the rebuttal letter that accompanies your revised manuscript. If you need to cite a retracted article, indicate the article’s retracted status in the References list and also include a citation and full reference for the retraction notice.

Additional Editor Comments:

Please try to improve the conclusion, I recommend to:

-Use more cautious language that reflects the limitations of the retrospective cross-sectional design.

-Provide more specific and actionable practice recommendations that are directly informed by the study findings.

-Explicitly acknowledge the potential impact of missing data and unmeasured confounding on the results.

-Clearly distinguish between findings observed in unadjusted analyses and those retained in the final multivariable model.

[Note: HTML markup is below. Please do not edit.]

To ensure your figures meet our technical requirements, please review our figure guidelines: https://journals.plos.org/plosone/s/figures

You may also use PLOS’s free figure tool, NAAS, to help you prepare publication quality figures: https://journals.plos.org/plosone/s/figures#loc-tools-for-figure-preparation.

NAAS will assess whether your figures meet our technical requirements by comparing each figure against our figure specifications.

# Author comment: Author response to Decision Letter 3

**DOI:** 10.1371/journal.pone.0351014.r007

19 Sep 2026

We sincerely thank the Academic Editor for the additional guidance regarding the Conclusion. We have carefully revised the Conclusion to address all four points raised in the latest editorial comments. We also reviewed the concerns raised by Reviewer #3 in the previous revision round and have ensured that these issues are now explicitly and consistently reflected in the revised Conclusion.

Comment 1

Academic Editor:

“Use more cautious language that reflects the limitations of the retrospective cross-sectional design.”

Response:

Thank you for this important comment. We revised the Conclusion to use more cautious language and to avoid causal interpretation. The revised text explicitly states that the retrospective cross-sectional design does not establish temporal or causal relationships. Throughout the Conclusion, we use association-based wording rather than causal language.

The revised text states:

“These findings should be interpreted cautiously because the retrospective cross-sectional design does not establish temporal or causal relationships, while missing clinical data and unmeasured disease severity may have influenced the observed associations.”

This revision also directly addresses the concern raised by Reviewer #3 in the previous revision round that some wording could imply stronger inference than supported by a retrospective cross-sectional analysis.

Comment 2

Academic Editor:

“Provide more specific and actionable practice recommendations that are directly informed by the study findings.”

Response:

Thank you for this recommendation. We have revised the practice implication in the Conclusion to provide a more specific recommendation that directly reflects the primary multivariable findings. Rather than providing a broad recommendation to monitor nutritional status, we now recommend routine nutritional screening during pediatric CHD follow-up, with particular attention to younger children and those with greater documented clinical burden.

The revised text states:

“In practice, routine nutritional screening during pediatric CHD follow-up, with particular attention to younger children and those with greater documented clinical burden, may facilitate earlier nutritional assessment and appropriate multidisciplinary management.”

This recommendation is directly informed by the observed association between younger age groups, higher cumulative clinical burden, and greater odds of malnutrition in the primary multivariable model. The cautious wording “may facilitate” avoids implying that the intervention itself has been demonstrated to improve clinical outcomes.

This revision also addresses Reviewer #3’s previous comment that the practice recommendations were broad and not sufficiently linked to specific actions.

Comment 3

Academic Editor:

“Explicitly acknowledge the potential impact of missing data and unmeasured confounding on the results.”

Response:

We agree with the Academic Editor. The revised Conclusion now explicitly acknowledges both missing clinical data and unmeasured disease severity as potential sources of uncertainty in the observed associations.

The revised text states:

“These findings should be interpreted cautiously because the retrospective cross-sectional design does not establish temporal or causal relationships, while missing clinical data and unmeasured disease severity may have influenced the observed associations.”

This wording reflects the limitations already described in the manuscript, including incomplete documentation of several clinical variables and the limited availability of detailed indicators of disease severity. We use “may have influenced” rather than attributing a specific direction or magnitude of bias because the available data do not permit us to determine the extent or direction of such effects.

This revision directly addresses Reviewer #3’s previous concern regarding uncertainty related to missing data and unmeasured confounding.

Comment 4

Academic Editor:

“Clearly distinguish between findings observed in unadjusted analyses and those retained in the final multivariable model.”

Response:

Thank you for highlighting this important distinction. We have revised the Conclusion to clearly separate the findings from the univariable analysis, the sensitivity analysis, and the primary multivariable model.

The revised Conclusion now states:

“Younger age groups and higher cumulative clinical burden were associated with greater odds of malnutrition in the primary multivariable model. Healthcare utilization was associated with malnutrition in univariable analysis and remained statistically significant in the sensitivity analysis, but it was not retained in the primary multivariable model.”

This wording makes clear that healthcare utilization was not an independent predictor retained in the primary multivariable model. In contrast, younger age groups and higher cumulative clinical burden represent the findings retained in the primary model.

This revision directly addresses Reviewer #3’s previous comment that the earlier Conclusion stated an association between malnutrition and increased healthcare utilization without sufficiently clarifying that this association was not demonstrated in the final primary multivariable model.

Clarification regarding the previous Reviewer #3 comments

We would also like to clarify that the four issues identified by the Academic Editor in the current revision correspond closely to the concerns previously raised by Reviewer #3 regarding the Conclusion. These concerns had been addressed during the previous revision, but we have further strengthened and made the relevant statements explicit in the Conclusion in response to the Academic Editor’s latest guidance.

Specifically, the revised Conclusion now explicitly addresses the four areas as follows:

Causal interpretation: We state that the retrospective cross-sectional design does not establish temporal or causal relationships.

Practice recommendations: We provide a specific recommendation for routine nutritional screening during pediatric CHD follow-up, particularly for younger children and those with greater documented clinical burden.

Missing data and unmeasured confounding: We explicitly acknowledge that missing clinical data and unmeasured disease severity may have influenced the observed associations.

Analytical distinction: We clearly distinguish the healthcare utilization finding from the univariable and sensitivity analyses and identify younger age and higher cumulative clinical burden as the findings retained in the primary multivariable model.

We have therefore revised the Conclusion to ensure that the final interpretation remains appropriately cautious, transparent, and directly aligned with the statistical analyses presented in the manuscript.

Revised Conclusion

The revised Conclusion reads:

“Pediatric outpatients with congenital heart disease at Adam Malik General Hospital had a substantial burden of malnutrition, with nearly half of the cohort classified as underweight or severely underweight. Younger age groups and higher cumulative clinical burden were associated with greater odds of malnutrition in the primary multivariable model. Healthcare utilization was associated with malnutrition in univariable analysis and remained statistically significant in the sensitivity analysis, but it was not retained in the primary multivariable model. These findings should be interpreted cautiously because the retrospective cross-sectional design does not establish temporal or causal relationships, while missing clinical data and unmeasured disease severity may have influenced the observed associations. In practice, routine nutritional screening during pediatric CHD follow-up, with particular attention to younger children and those with greater documented clinical burden, may facilitate earlier nutritional assessment and appropriate multidisciplinary management. Prospective studies using standardized nutritional and clinical severity measures are needed to confirm these associations.”

We appreciate the Academic Editor’s guidance, which has helped us further improve the precision and transparency of the Conclusion. We believe that the revised version now clearly distinguishes the primary multivariable findings from secondary analyses, appropriately acknowledges the study limitations, and provides practice implications that are directly informed by the study findings.

Attachment Submitted filename: Response to academic editor.docx

# Editor report: Decision Letter 3

**DOI:** 10.1371/journal.pone.0351014.r008

**Authors:** Hany Mahmoud Abo-Haded

23 Sep 2026

Nutritional status, clinical burden, and healthcare utilization among pediatric outpatients with congenital heart disease: A retrospective cross-sectional study from Indonesia

PONE-D-26-24373R3

Dear Dr. Amelia,

We’re pleased to inform you that your manuscript has been judged scientifically suitable for publication and will be formally accepted for publication once it meets all outstanding technical requirements.

Within one week, you’ll receive an e-mail detailing the required amendments. When these have been addressed, you’ll receive a formal acceptance letter and your manuscript will be scheduled for publication.

An invoice will be generated when your article is formally accepted. Please note, if your institution has a publishing partnership with PLOS and your article meets the relevant criteria, all or part of your publication costs will be covered. Please make sure your user information is up-to-date by logging into Editorial Manager at Editorial Manager® and clicking the ‘Update My Information' link at the top of the page. For questions related to billing, please contact billing support.

If your institution or institutions have a press office, please notify them about your upcoming paper to help maximize its impact. If they’ll be preparing press materials, please inform our press team as soon as possible -- no later than 48 hours after receiving the formal acceptance. Your manuscript will remain under strict press embargo until 2 pm Eastern Time on the date of publication. For more information, please contact onepress@plos.org.

Kind regards,

Hany Mahmoud Abo-Haded, MD

Academic Editor

PLOS One

Additional Editor Comments (optional):

Reviewers' comments:

# Editor report: Acceptance letter

**DOI:** 10.1371/journal.pone.0351014.r009

**Authors:** Hany Mahmoud Abo-Haded

PONE-D-26-24373R3

PLOS One

Dear Dr. Amelia,

I'm pleased to inform you that your manuscript has been deemed suitable for publication in PLOS One. Congratulations! Your manuscript is now being handed over to our production team.

At this stage, our production department will prepare your paper for publication. This includes ensuring the following:

* All references, tables, and figures are properly cited

* All relevant supporting information is included in the manuscript submission,

* There are no issues that prevent the paper from being properly typeset

You will receive further instructions from the production team, including instructions on how to review your proof when it is ready. Please keep in mind that we are working through a large volume of accepted articles, so please give us a few days to review your paper and let you know the next and final steps.

Lastly, if your institution or institutions have a press office, please let them know about your upcoming paper now to help maximize its impact. If they'll be preparing press materials, please inform our press team within the next 48 hours. Your manuscript will remain under strict press embargo until 2 pm Eastern Time on the date of publication. For more information, please contact onepress@plos.org.

You will receive an invoice from PLOS for your publication fee after your manuscript has reached the completed accept phase. If you receive an email requesting payment before acceptance or for any other service, this may be a phishing scheme. Learn how to identify phishing emails and protect your accounts at https://explore.plos.org/phishing.

If we can help with anything else, please email us at customercare@plos.org.

Thank you for submitting your work to PLOS One and supporting open access.

Kind regards,

PLOS One Editorial Office Staff

on behalf of

Professor Hany Mahmoud Abo-Haded

Academic Editor

PLOS One
